# Membrane damage induces TagQ/TagR complex-mediated H1-T6SS assembly in *Pseudomonas aeruginosa*

**DOI:** 10.1038/s41467-026-78112-9

**Published:** 2026-09-28

**Authors:** Maxim Kolesnikov, Karolin Berneiser, Pradyot Prakash, Marek Basler

**Affiliations:** Biozentrum, University of Basel, Basel, Switzerland

**Keywords:** Pathogens, Biochemistry, Bacterial genetics, Bacterial secretion

## Abstract

The type VI secretion system (T6SS) is a large macromolecular complex crucial for inter-bacterial competition. Its expression and localization are tightly regulated, with the anti-bacterial H1-T6SS in *Pseudomonas aeruginosa* assembling in response to membrane damage. While several proteins are known to regulate this response, the mechanism remains unknown. Here, we use biochemical approaches to show formation of a complex between the membrane lipoprotein TagQ and the periplasmic protein TagR. Furthermore, Co-IP and in silico modeling indicates TagQ/R complex interactions with PpkA, likely dimerizing the kinase to initiate a phosphorylation cascade leading to H1-T6SS assembly. Mass spectrometry analysis of phosphorylated proteins in *P. aeruginosa* shows that PpkA does not have any targets outside of the previously reported Fha1, suggesting the sensor module acts exclusively to regulate T6SS assembly. Our work reveals the functional basis of the TagQR-PpkA signaling pathway and links membrane damage to localized H1-T6SS assembly, which is critical for efficient retaliation during inter-bacterial interactions.

## Abstract (web summary)

The H1-T6SS of *Pseudomonas aeruginosa* assembles in response to membrane damage. Here, the authors show that this response mechanism is regulated by the formation of a complex between the membrane lipoprotein TagQ and the periplasmic protein TagR.

## Introduction

Competition for a limited set of resources between bacteria requires the ability to manipulate the immediate environment of the bacterial cell in response to a wide range of external stimuli. For this purpose, bacteria use large macromolecular complexes called secretion systems to secrete a variety of small molecules and proteins into the environment as well as directly to the cytoplasm of both prokaryotic and eukaryotic cells^1,2^. One of the main secretion systems used for interbacterial competition is the well-described type VI secretion system (T6SS), present in a large subset of Gram-negative bacteria^3,4^.

While the overall structure and function of the T6SS is relatively conserved across different species, the mechanism by which assemblies are localized and initiated can vary significantly. In some species, such as *Vibrio cholerae* T6SS structures are randomly distributed^5,6^. Alternatively, *Francisella novicida* and *Burkholderia thailandensis*, both display a polar localization preference for their T6SS^7–9^. The presence of neighboring cells can also affect T6SS localization, as *Acinetobacter* was shown to assemble up to half of their assemblies at contact sites between neighboring cells^10^. Uniquely among other species, *Pseudomonas aeruginosa*, which expresses three different T6SS encoded on separate gene clusters, uses its main anti-bacterial H1-T6SS solely as a counterattack mechanism. H1-T6SS is assembled in very close proximity to membrane damage, which results in a characteristic “tit-for-tat” phenotype where *P. aeruginosa* cells respond to incoming T6SS attacks. Similarly, *P. aeruginosa* cells are able to “duel” in a prolonged manner with their T6SS, as they are immune to their own effectors^11,12^. Recent work showed that dueling can be initiated by sister cells initiating a T6SS attack using the H2-T6SS^13^. Additionally, assembly can also be induced by other means, such as polymyxin B, chelation of ions, extracellular DNA, or other secretion systems, such as the type IV secretion system^11,12,14,15^. Penetration of the outer membrane alone is sufficient to trigger the response, as determined using atomic force microscopy^16^.

The regulation of H1-T6SS assembly has been shown to depend on a threonine phosphorylation pathway (TPP) consisting of the inner membrane type II Hanks-type threonine kinase PpkA and the protein phosphatase 2C (PP2C) family phosphatase PppA, as well as a putative sensor module consisting of the proteins TagQ/TagR/TagS and TagT acting upstream of kinase activation^17–19^.

The outer membrane (OM), 31.7 kDa lipoprotein TagQ is anchored to the inner leaflet via a lipidation of the conserved cysteine 30 residue within a lipobox sequence recognized by the inner membrane signal peptidase II^18,20^. Aside from being localized to the OM, TagQ is strikingly abundant at nearly 61,000 copies per cell^21^. Subcellular fractionation, as well as fluorescence microscopy previously showed that TagQ co-localizes to the outer membrane with the 62 kDa periplasmic protein TagR, itself far less abundant at only around 1000 copies^18,21^. However, while TagQ is a perfect candidate for signal detection, no direct evidence of interactions with either TagR or other proteins has been reported, and it remains uncertain what role TagQ plays in the sensor module.

Both TagQ and TagR were found to act upstream of the 110 kDa inner membrane threonine kinase PpkA. PpkA was shown to autophosphorylate upon dimerization and initiate H1-T6SS assembly by phosphorylation of the cytoplasmic protein Fha1 in an oligomerization dependent manner. The function of PpkA is antagonized by the inner membrane phosphatase PppA, which dephosphorylates Fha, and the deletion of which results in disturbed localization of H1-T6SS and prolonged presence of assemblies. Autophosphorylation of PpkA requires TagR, as a Δ*tagR* mutant is defective in Hcp1 secretion, and depletion of TagR results in a significant reduction of phosphorylated PpkA. However, although TagR activity has been linked to both the outer membrane and inner membrane components of the H1-T6SS damage response mechanism, whether it interacts directly with PpkA to promote dimerization remains unknown^17–19^.

The final component of the sensor module is the TagS/TagT inner membrane complex, which shares structural similarity with ABC transporters. While it is indispensable for the membrane damage response and has been shown to have ATPase activity in vitro, its role is the least defined, with no known functional link to other sensor module components^18,22–24^. Existing models of H1-T6SS assembly have proposed that activation of PpkA by TagR is dependent on interactions with both TagQ and the TagS/TagT complex^18,19^. However, the lack of data for direct binding between any of the proteins outside of the TagS/T complex itself means that the molecular mechanism for how a signal is carried from the outer membrane of *P. aeruginosa* to the inner membrane remains largely undefined.

In this study, we aimed to describe the molecular mechanism of signal transduction from the OM of *P. aeruginosa* to PpkA, through the TagQRST sensor module. Using several biochemical approaches, we found that the outer membrane lipoprotein TagQ forms a stable 2:2 complex with TagR. We further show that TagR is able to dimerize in the absence of TagQ, albeit at supraphysiological concentrations. In contrast, we were unable to show any stable interaction between TagS/T and other sensor module components, although its ATPase activity is required in vivo for sensor module function. Importantly, using several different molecules, we show that membrane damage promotes TagQ/R complex formation. Moreover, we also detect the formation of a ternary complex between TagQ, TagR, and PpkA. This, combined with in silico modeling, suggests that the dimerization of PpkA is mediated via binding of two periplasmic domains of PpkA to a TagQ/R complex. Finally, we show by mass spectroscopy of phosphorylated proteins that PpkA exclusively phosphorylates Fha1 to activate H1-T6SS assembly. Based on these findings, we propose a model of H1-T6SS activation in *P. aeruginosa,* which represents a unique membrane damage response mechanism in Gram-negative bacteria.

## Results

### TagQ forms a 2:2 complex with TagR

**Fig. 1:**
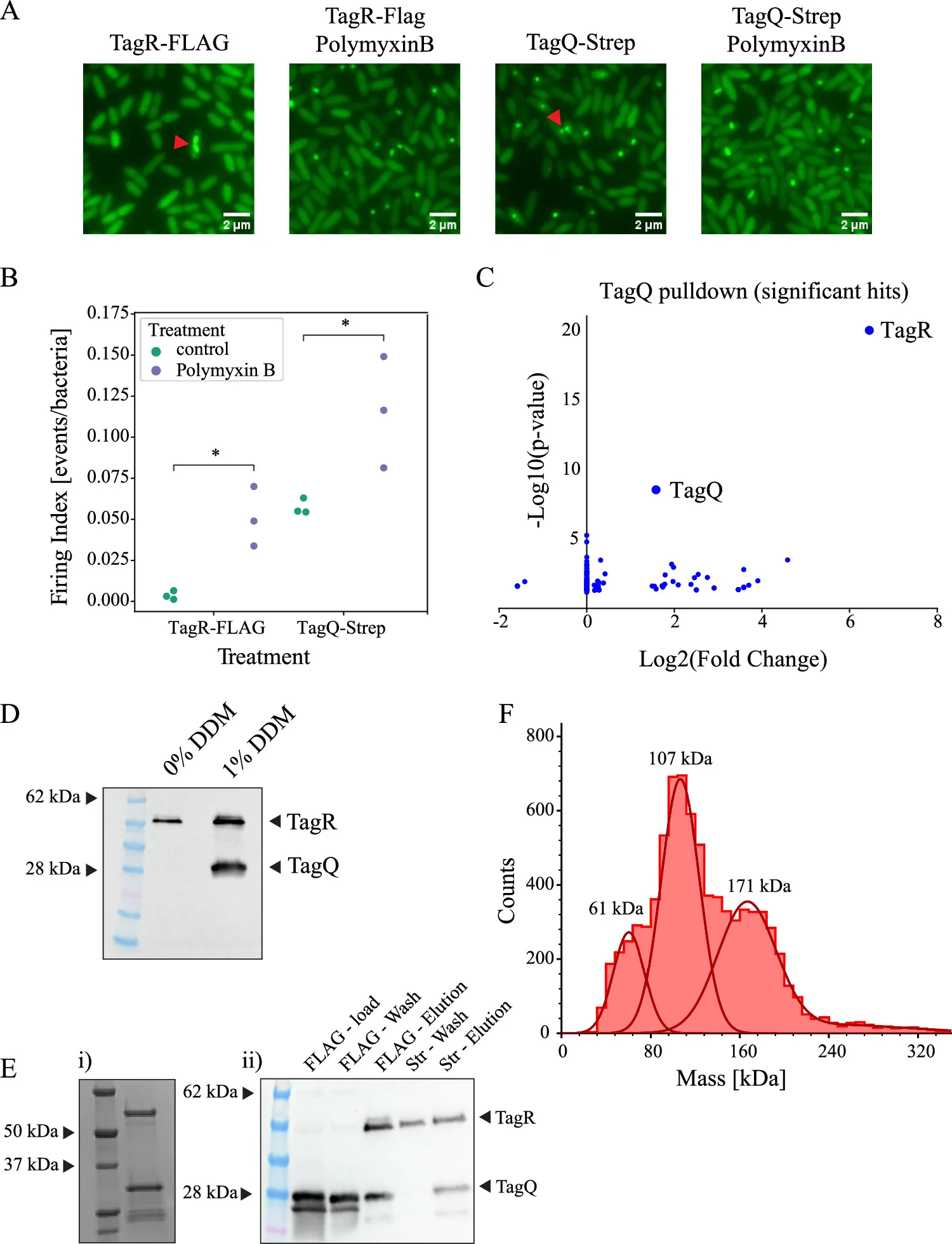
TagQ and TagR form a complex in vivo. **A** Live-cell microscopy snapshots showing dueling events indicated with red markers and response to polymyxin B for TagR-FLAG and TagQ-Strep strains. **B** Independent *t*-test statistical analysis of firing events per bacterium on a single frame for TagR-FLAG (*tagR-FLAG ΔretS tssB1-mNG*) and TagQ-Strep (*tagQ-Strep ΔretS tssB1-mNG*) strains. **C** Significant hits for peptides enriched in TagQ-FLAG Co-IP experiment vs WT strain. Two-tailed unpaired *t*-test. Resulting p-values are displayed, values below p-value threshold of 0.05 omitted. **D** Representative western blot with anti-TagQ and anti-TagR antibodies showing co-purification of TagR-FLAG and TagQ from *P. aeruginosa* with or without the presence of DDM in the lysis buffer. Experiment performed in triplicate. **E** Representative SDS-PAGE gel (i) and western blot using anti-TagQ and anti-TagR antibodies (ii) of TagQR complex purified by tandem affinity purification using anti-FLAG and anti-Strep beads. Experiment performed in triplicate **F** Microfluidic mass photometry analysis of TagQR purified by tandem affinity showing aggregate counts as histograms with mass estimates derived from fitted normal distributions. Three populations can be identified with estimated masses of 61, 107, and 171 kDa respectively. ∗ = *p* < 0.05. Two-sided independent Student’s *t*-test. All exact p-values available in supplementary table of Supplementary dataset 7.

To determine whether the function of TagQ required interactions with other sensor module components, we first cloned affinity tags directly on the chromosome of *P. aeruginosa*. To test sensor module function, all cloning with affinity tags attached to TagQ or TagR was performed in a strain lacking *retS* to increase H1-T6SS expression and expressing the sheath protein TssB1 fused to mNeonGreen, allowing for the visualization of H1-T6SS sheath assembly and contraction. These strains were observed by live-cell fluorescence microscopy for the presence of dueling and induction of H1-T6SS activity via polymyxin B, a small peptide causing extensive membrane damage^14^. H1-T6SS activity and response to polymyxin B of C-terminal fusion constructs TagR-FLAG and TagQ-Strep were comparable to the parental strain (Fig. 1A, B and Supplementary Video 1). To test whether the addition of affinity tags to both proteins may impact in vivo function and subsequently purification, we performed a killing assay comparing our experimental strains to the parental phenotype. This showed T6SS-dependent killing of prey *A. baylyi* ADP1 cells with no difference in killing efficiency (Fig. S2).

To identify proteins interacting with TagQ, we performed Co-immunoprecipitation (Co-IP) with *tagQ-FLAG ΔretS tssB1-mNG* strain lysate incubated with anti-FLAG beads, followed by mass spectrometry (MS) of the eluate, which resulted in significant enrichment of TagR when compared to a *ΔretS tssB1-mNG* control strain (Fig. 1C and Table S1).

To further confirm the interaction of TagQ and TagR, we performed further Co-IPs using both *tagQ-FLAG* Δ*retS tssB1-mNG* and *tagR-FLAG* Δ*retS tssB1-mNG* strains. TagQ and TagR consistently co-purified in the presence of detergent, as assayed by western blots using anti-TagQ and anti-TagR antibodies (Fig. 1D). We were able to purify the complex using a tandem affinity approach and confirm purity and identity using SDS-PAGE, western blot, and MS analysis (Fig. 1E). A lower sized band was identified by MS analysis as TagQ with partial N-terminal degradation. Interestingly, size exclusion chromatography (SEC) analysis of proteins purified using anti-FLAG beads showed a mixed population across a wide size range, with several possible stoichiometries for the complex (Fig. S1A, B). To validate this estimate, we used mass photometry to measure the mass of the purified complex. Analysis of the samples identified three distinct populations of 61, 107, and 171 kDa. Assuming the purified proteins no longer have a signal peptide sequence, as seen in our MS analysis of the samples, these weights correspond to a TagR monomer (59 kDa), TagR dimer (118 kDa), and a complex with a 2:2 stoichiometry (176 kDa) (Fig. 1F). Unfortunately, the complex quickly dissociated under sample conditions required for mass photometry, revealing degradation of the complex to a monomeric TagR state within 1 min of sample mixing (Fig. S1C, D).

Anchoring of TagQ to the OM is achieved through a lipidation of a conserved cysteine residue in a lipobox sequence near the N-terminus of the protein^18^. As such, we wondered if removal of the lipidation would affect TagQ/TagR complex formation. Using live-cell fluorescence microscopy, we were able to confirm that a C30A mutant is deficient in H1-T6SS dueling and responding to polymyxin B treatment, however, this mutant was still able to form a complex with TagR as assayed by western blot (Fig. S1E, F). Together, these data indicate that TagQ forms a 2:2 complex with TagR. Additionally, complex formation is independent of the lipidation of TagQ.

### TagR forms a homodimer at supraphysiological concentrations

**Fig. 2:**
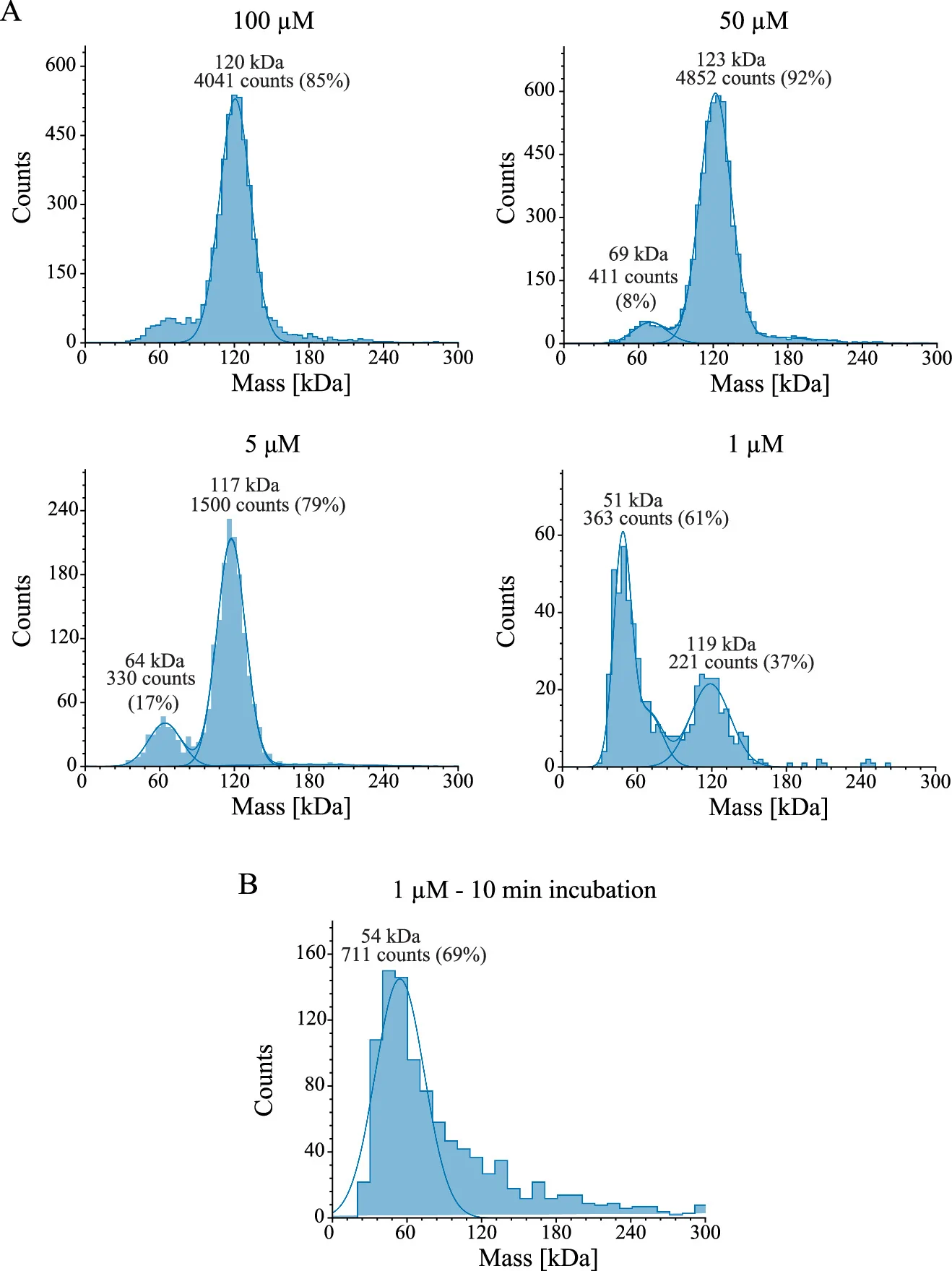
TagR forms a dimer in vitro at supraphysiological concentrations. **A** Mass photometry analysis of TagR at different concentrations shows predominance of dimer population (118 kDa) above 5 μM and a majority monomeric (59 kDa) population at 1 μM. **B** Incubation of TagR at 1 μM for 10 min after dilution produces a population that does not have a distinct dimer peak. Aggregate counts are shown as histograms with mass estimates derived from fitted normal distributions.

As the stoichiometry of the TagQ/TagR complex was measured to be 2:2, we wondered if the oligomeric state of TagR was influenced by TagQ binding. For this, we purified TagR directly from *P. aeruginosa*, TagQ contamination was avoided by removing detergent from the purification protocol and assessing sample purity by SDS-PAGE and western blotting (Fig. S3A, B). Subsequently, the oligomeric state of TagR was assayed using mass photometry. Interestingly, analysis showed that TagR is able to form homodimers in vitro in the absence of TagQ (Fig. 2A). In particular, TagR exists in a predominantly dimeric state above concentrations of 5 μM and quickly dissociates into a monomer upon further dilution (Fig. 2A). As reported previously, there are about 1000 copies of TagR in the cell^21^. Considering an estimated periplasmic volume in *P. aeruginosa*, we calculated the in vivo concentration of TagR to be approximately 830 nM^25^. Mass photometry measurements of TagR at 1 μM showed a population mostly in a monomeric state (Fig. 2A, B). These results suggest that although TagR is capable of homodimerization in the absence of other proteins, it does this in vitro at supraphysiological concentrations.

### Cryo-EM analysis suggests TagR forms a symmetrical dimer

**Fig. 3:**
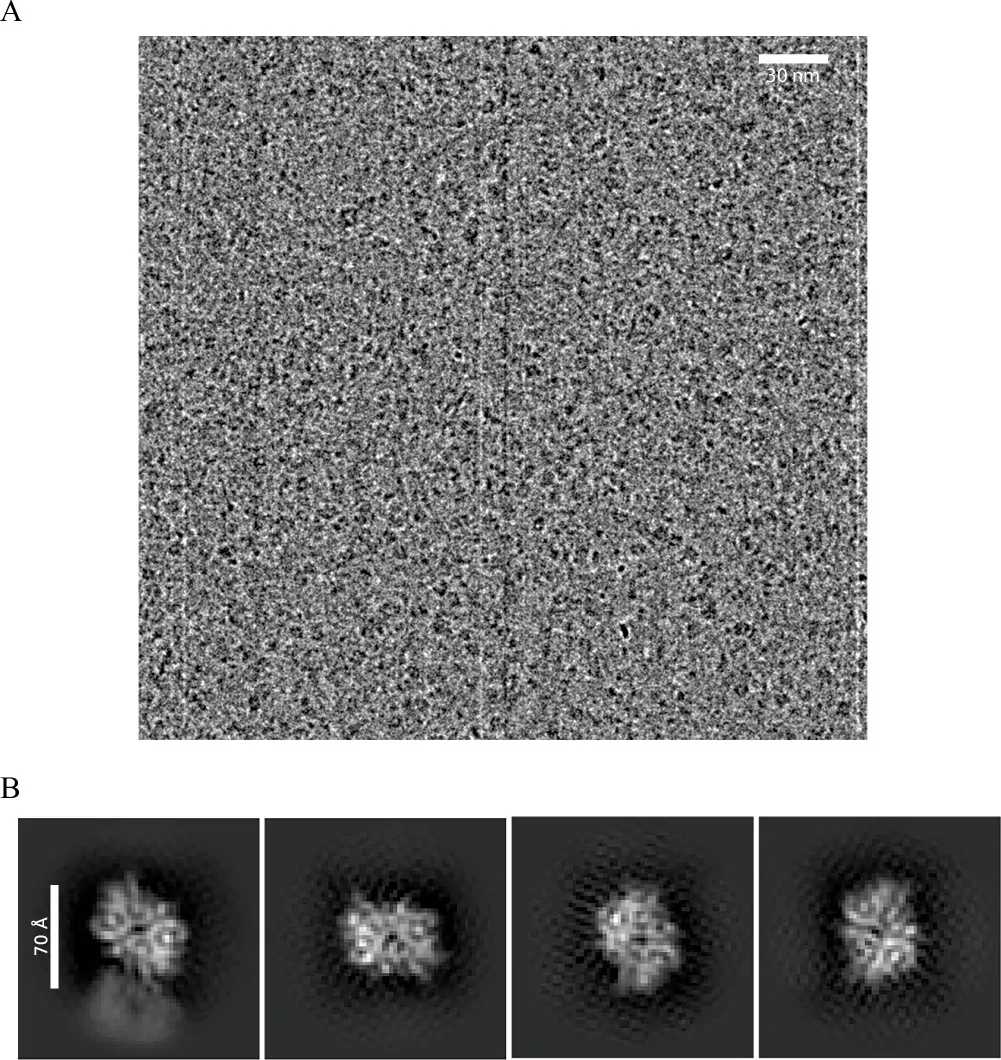
Cryo-EM of TagR indicates the TagR dimer is a flexible symmetrical structure. **A** Raw micrograph of TagR samples on Quantifoil R1.2/1.3 holey carbon copper grids (Quantifoil Micro Tools). Data were collected using a Titan Krios (Thermo Fisher Scientific FEI) transmission electron microscope equipped with a K2 Summit direct electron detector (Gatan) using SerialEM. **B** Representative 2D classes of TagR samples on Quantifoil R1.2/1.3 holey carbon copper grids (Quantifoil Micro Tools). Data were collected using a Titan Krios (Thermo Fisher Scientific FEI) transmission electron microscope equipped with a K2 Summit direct electron detector (Gatan) using SerialEM.

To obtain further information about the TagQ/TagR complex, we initially attempted several approaches to express both proteins recombinantly. While we were able to purify both proteins, we were unable to reconstitute the complex or obtain sufficient yields (Fig. S4A–C). Instead, we shifted to working with native purifications from *P. aeruginosa*. To determine the structure of the TagR dimer, TagR samples were purified from *P. aeruginosa*, applied to carbon copper grids and plunge frozen at concentrations of approximately 3 mg/ml (48 µM). Data collection on a Titan Krios with a K2 camera revealed raw micrographs and 2D classes following processing in cryoSPARC which showed particles of around 70–80 Å with C2 symmetry (Fig. 3A, B and Table S4). This size aligns with an AlphaFold3 model of a TagR dimer (Fig. S6). Unfortunately, multiple rounds of 2D and 3D classification failed to converge to a high-resolution map likely due to an overall flexibility and heterogeneity of the particles. This suggests that TagR has a high degree of flexibility even as a dimer. Similarly, we attempted to obtain the cryo-EM structure of the TagQ/TagR complex, however, these particles displayed a high degree of heterogeneity, which made 3D reconstruction not possible. In summary, these data support our observations of TagR homodimerization at high concentrations.

### TagST ATPase activity is required for sensor module function

**Fig. 4:**
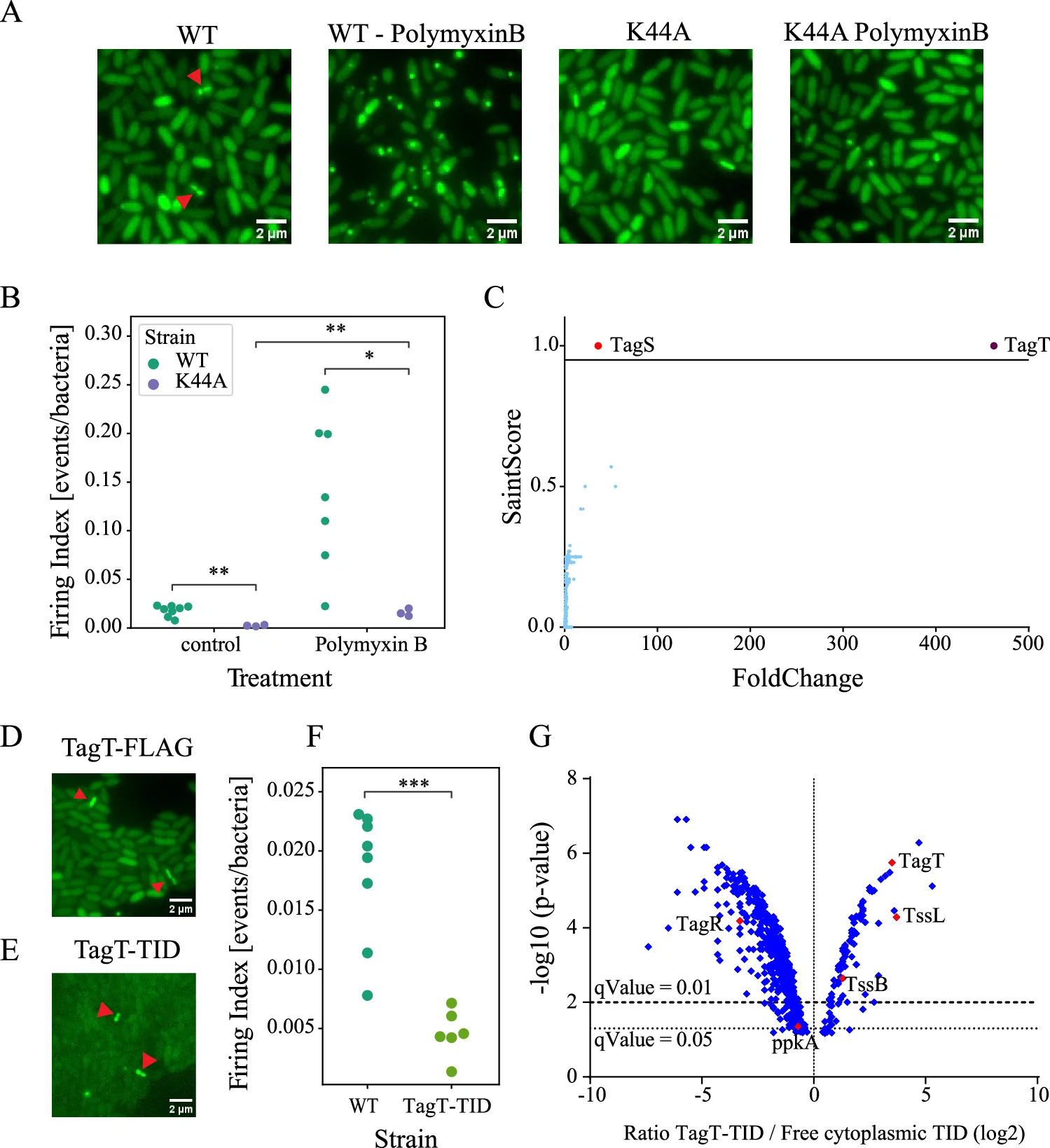
TagST ATPase activity is required for sensor module function. **A** Live-cell imaging of WT (*ΔretS tssB1-mNG*) and TagT K44A (*tagT-K44A ΔretS tssB1-mNG*) cells with or without polymyxin B stimulation. Dueling events indicated with red markers. **B** Independent *t*-test statistical analysis of firing events per bacterium on a single frame. **C** SAINT score (Choi et al., 2011) MS analysis of peptides enriched in a TagT-FLAG Co-IP vs WT strain, top hits (CI > 95%) colored red and purple. **D** Live microscopy imaging showing TagT-FLAG construct with dueling events indicated with red markers. **E** Live microscopy imaging inset showing TagT-TID construct with dueling events indicated with red markers. **F** Independent *t*-test statistical analysis of firing events per bacterium on a single frame for WT (*ΔretS tssB1-mNG*) and TagT-TurboID (*tagT-TurboID ΔretS tssB1-mNG*) cells. Microscopy inset showing dueling events identified by a red marker in the TagT-TurboID strain. *p*-values: ∗ = *p* < 0.05, ∗∗ = *p* < 0.01, ∗∗∗ = *p* < 0.001. Two-sided independent Student’s *t*-test. All exact *p*-values available in the Supplementary Table of Supplementary dataset 7. **G** Volcano plot of significant hits from MS analysis of peptides identified in a TurboID pulldown experiment. Empirical Bayes moderated *t*-test; *p*-values were adjusted for multiple testing using the Benjamini-Hochberg method (resulting *q*-values are displayed).

Aside from TagQ and TagR, the sensor module contains an ABC-transporter like complex consisting of the proteins TagS and TagT located in the inner membrane. It was shown previously that *tagT* and *tagS* deletion strains secrete less Hcp1 and display a loss of H1-T6SS mediated killing of prey cells. Additionally, the TagST complex is capable of ATP hydrolysis in vitro^18^. To investigate whether ATP hydrolysis was required for H1-T6SS functions in vivo, we generated a mutant strain (*tagT K44A ΔretS tssB1-mNG*) with the same K44A mutation in the conserved Walker A region of TagT that was reported to completely halt ATP hydrolysis. Visualization of H1-T6SS assembly in this mutant by live-cell microscopy showed a significantly lower level of H1-T6SS activity than a parental strain. Moreover, no T6SS dueling with sister cells and no response to polymyxin B stimulation were detected (Fig. 4A, B and Supplementary Video 2). Taken together, we found that the TagS/TagT complex requires active ATP hydrolysis to support H1-T6SS activation in vivo.

### TagST does not pull down with sensor module components

Given that the existing models of H1-T6SS activation propose direct interactions between TagS/TagT and other components of the sensor module^18^, we attempted to identify potential binding partners using Co-IP approaches. A PAO1 *ΔretS tssB1-mNG tagT-FLAG* mutant strain was generated and examined by live-cell microscopy. No difference in dueling frequency was observed relative to the parental strain (Fig. 4D and Supplementary Video 1). Co-IP using lysate from this strain incubated with anti-FLAG beads, followed by MS analysis of enriched peptides, found that only TagS and TagT were significantly enriched in comparison to an untagged control (PAO1 *ΔretS tssB1-mNG*) (Fig. 4C and Table S2).

Considering that Co-IP analysis requires relatively stable complexes that do not dissociate during the purification process, we attempted to identify the in vivo interactome of the TagS/TagT complex using a proximity labeling approach based on the TurboID system, which biotinylates proximal proteins using a modified biotin ligase attached to a protein of interest^26^. For this, we generated a strain (*ΔretS tssB1-mNG tagT-TurboID)* containing the TurboID biotin ligase as a fusion protein on the C-terminal end of TagT, which is exposed to the cytoplasm, as the system cannot be used in the periplasm due to a lack of ATP. We confirmed that H1-T6SS activity and dueling remained comparable to the parental strain (Fig. 4E, F and Supplementary Video 1). Similarly, a killing assay showed that killing of prey *A. baylyi* ADP1 cells remained T6SS-dependent and was comparable to the parental strain.

MS analysis of enriched proteins, compared to a control expressing TurboID as a soluble protein in the cytoplasm, failed to identify any sensor module components as potential binding partners (Fig. 4G and Table S3). However, several structural components, including the membrane complex protein TssL and sheath protein TssB, were enriched (Fig. 4G and Table S3).

Taken together, our data suggest that the TagS/TagT complex does not interact with other components of the sensor module, although its interactome may include other T6SS components.

### TagQR complex formation is induced by membrane damage

**Fig. 5:**
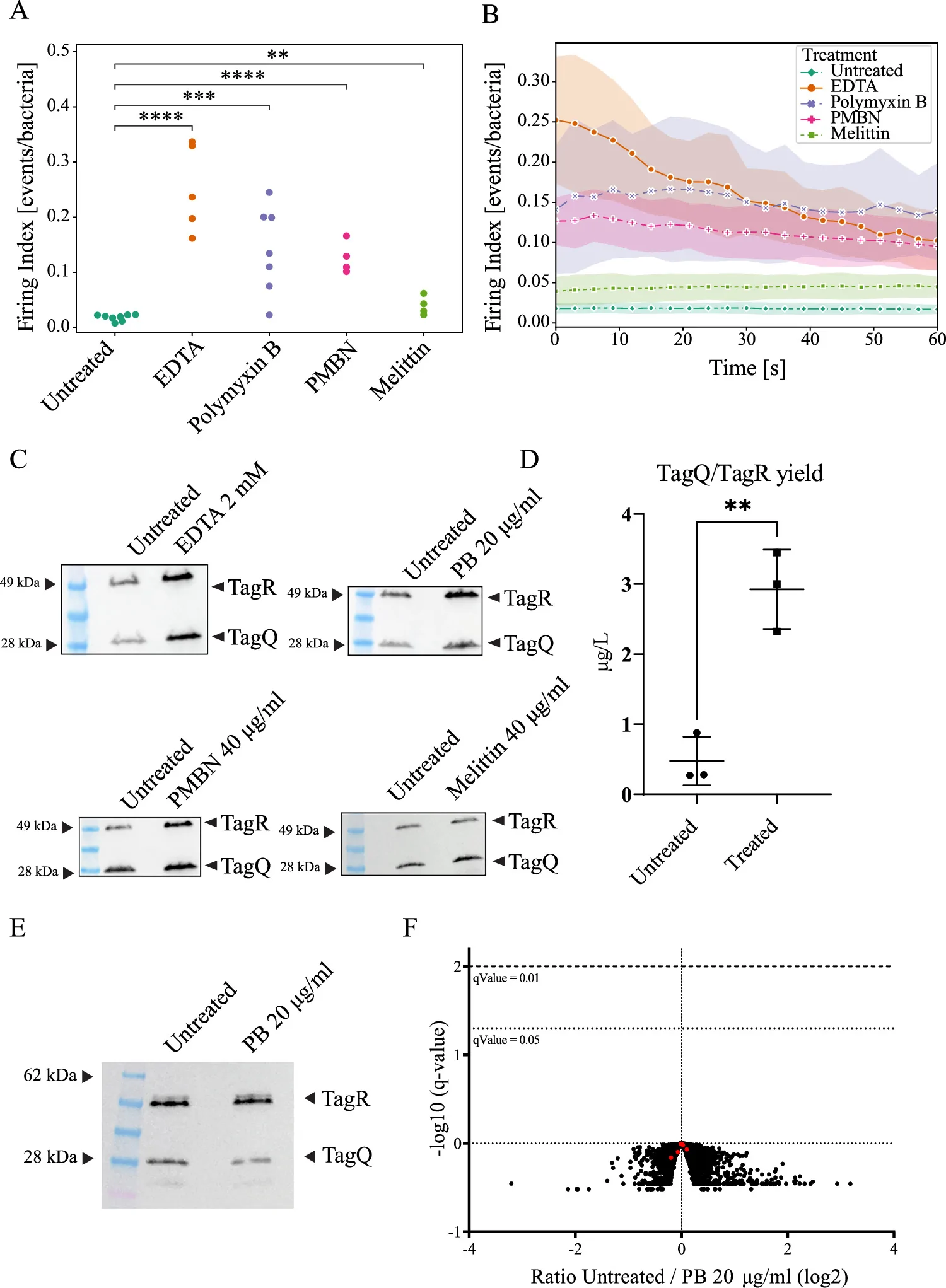
TagQR complex formation is induced by different sources of membrane damage. **A** Independent *t*-test statistical analysis of live-cell imaging firing events per bacterium on a single frame for *ΔretS tssB1-mNG P. aeruginosa* cells either untreated or treatment added to the LP agar pad. **B** Graphical representation of firing events (mean with shaded standard deviation) per bacterium over a 1 min interval with different applied treatments. **C** Western blots using anti-TagQ and anti-TagR antibodies of tandem purified TagQR complex from *P. aeruginosa* cells that were either treated or untreated prior to lysis. **D** Scatter dot plot of three separate biological replicates per condition (line at mean with standard deviation) showing yields in µg/L of TagQ/TagR complex purified directly from *P. aeruginosa* cells (unpaired Welch’s *t*-test, *p*-value 0.0057). Cells were left untreated or treated with 20 µg/ml polymyxin B immediately prior to lysis. **E** Western blot using anti-TagQ and anti-TagR antibodies comparing yield of purified complex with or without treatment of lysate with 20 µg/ml polymyxin B. **F** Volcano plot of hits from MS analysis of peptides identified in whole cell *P. aeruginosa* proteome, comparing abundance in untreated cells with cells treated with 20 µg/ml polymyxin B prior to lysis. Sensor module proteins colored red. Treatment concentrations for all experiments: EDTA 2 mM, Polymyxin B 20 μg/ml, PMBN 40 μg/mL, Melittin 40 μg/ml *p*-values: ∗ = *p* < 0.05, ∗∗ = *p* < 0.01, ∗∗∗ = *p* < 0.001, ∗∗∗∗ = *p* < 0.0001. Two-sided independent Student’s *t*-test. All exact *p*-values available in supplementary table of Supplementary dataset 7.

Having detected the formation of a complex between TagQ and TagR, we wondered if this occurs as part of the response to membrane damage. To investigate this, we used a combination of treatments previously known to trigger H1-T6SS assembly, such as polymyxin B and EDTA; as well as polymyxin B nonapeptide (PMBN), a truncated variant of polymyxin B acting only at the OM, and melittin, a component of bee venom previously reported to disrupt OM structure in Gram-negative bacteria^27,28^. Live-cell fluorescence imaging of parental strain on agar pads containing these compounds confirmed that all four were able to induce H1-T6SS assembly (Fig. 5A, B and Supplementary Video 3). Using TagQ-Strep TagR-FLAG strain, we assayed complex formation immediately following treatment of *P. aeruginosa* cells. Cells were treated with one of the target compounds, lysed immediately afterwards, and TagQR complex was purified using tandem affinity purification. Analysis of TagQR abundance using western blotting with specific antibodies showed an increase in purified complex for all samples except melittin when compared to untreated samples (Fig. 5C). To quantify the increase in yield, we performed several large-scale purifications either with or without pretreatment of cells using polymyxin B immediately prior to lysis. Yields increased significantly when cells were treated, by an average of 6-fold (Fig. 5D). To confirm that increased yields were not dependent on a solubilisation effect conveyed by polymyxin B itself, we performed a purification of the complex with polymyxin B added after cell lysis which did not result in increased yields (Fig. 5E). Additionally, to test whether the increase was due to protein production prior to cell death, we used MS to compare the abundance of all sensor module proteins following polymyxin B treatment when compared to mock treated samples. Analysis of peptide abundance showed no differences between either sample (Fig. 5F).

In summary, these results suggest that damage to the membrane, whether both at the OM and IM or at the OM alone, promotes formation of the TagQR complex.

### PpkA phosphorylation is limited to Fha1

**Fig. 6:**
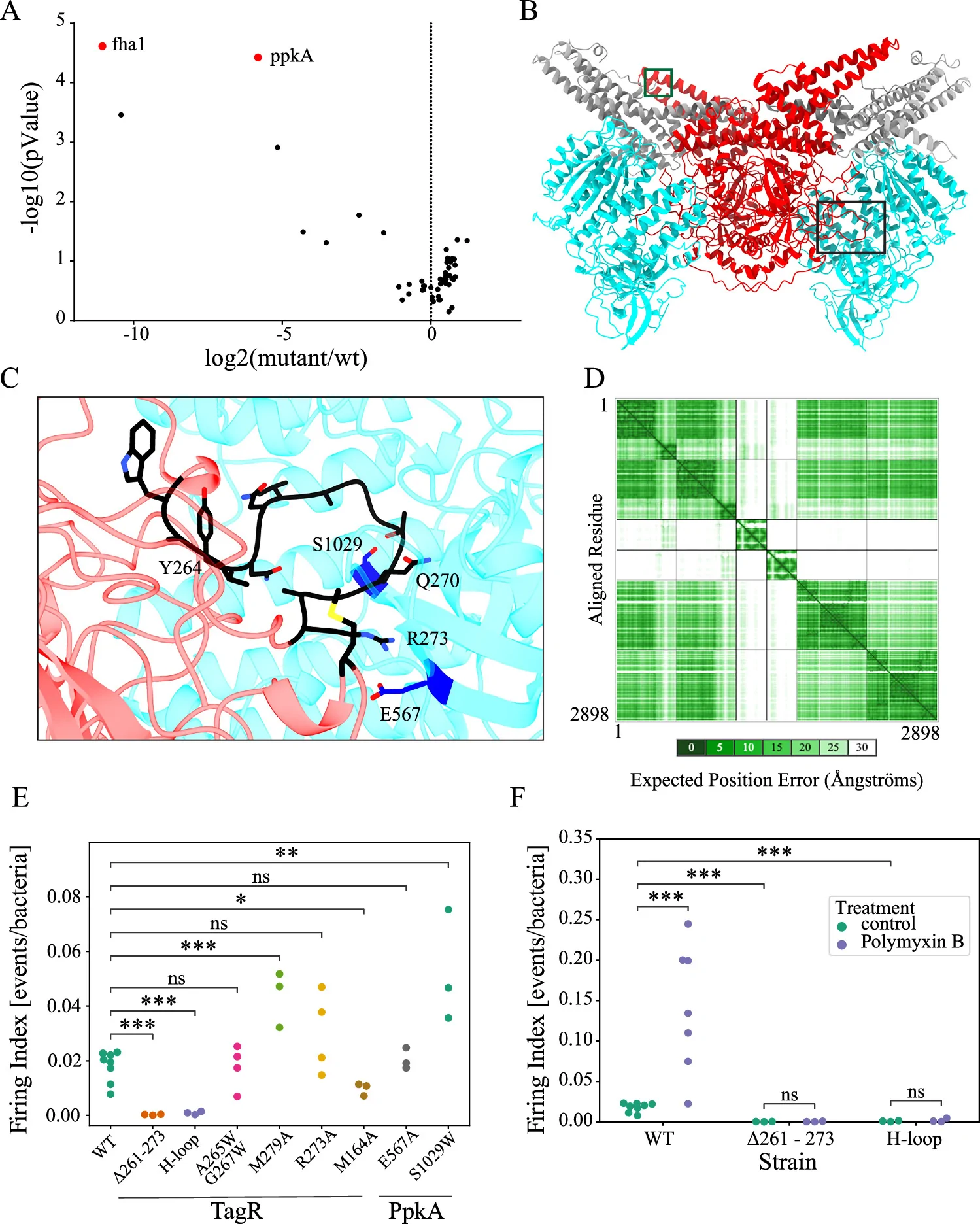
PpkA is predicted to interact with TagQ/TagR. **A** PhosphoMS analysis comparing abundance of purified phosphorylated peptides from WT or ΔPpkA *P. aeruginosa* strains. Highest probability hits colored red. Duplicate peptide hits removed. Empirical Bayes moderated *t*-test. Resulting p-values are displayed. **B** AlphaFold3 model of a TagR (red) – TagQ (grey) – PpkA (cyan) complex showing a symmetrical 2:2:2 stoichiometry. TagQ N-terminus highlighted by green box, TagR loop at a binding interface with PpkA highlighted by black box. **C** Zoomed in view of 12 amino acid loop of TagR (black) predicted to interact with a corresponding pocket in PpkA, forming part of the binding interface. **D** Predicted aligned error plot of AlphaFold3 model. ipTM = 0.65, pTM = 0.68. **E** Independent *t*-test statistical analysis of live-cell imaging firing events per bacterium on a single frame for WT, TagR mutant, and PpkA mutant *P. aeruginosa* strains. H-loop represents the mutations R273A, Q270A, T268A, Q263A, N262A, Y261A. **F** Independent *t*-test statistical analysis of live-cell imaging firing events per bacterium on a single frame for WT, loop deletion, and H-loop *P. aeruginosa* strains. *p*-values: ∗ = *p* < 0.05, ∗∗ = *p* < 0.01, ∗∗∗ = *p* < 0.001, ∗∗∗∗ = *p* < 0.0001. Two-sided independent Student’s *t*-test. All exact *p*-values are available in the Supplementary Table of Supplementary dataset 7.

Activation of H1-T6SS assembly proceeds through the IM kinase PpkA, which phosphorylates Fha1, promoting the assembly of other structural components^19^. However, as the possible targets of PpkA were not identified systematically, we chose to characterize the entire phosphoproteome of PpkA. For this, day-cultures of both parental and *ΔppkA ΔretS tssB1-mNG* strains were grown to exponential phase, and phosphopeptides were enriched by affinity chromatography. Analysis of the enriched peptides in the parental strain showed significant enrichment of PpkA and Fha1 when compared to the Δ*ppkA* control (Fig. 6A). These data suggest that PpkA does not have phosphorylation targets outside of Fha1 and is restricted to regulation of H1-T6SS assembly.

### AlphaFold modeling predicts a TagQ/TagR complex with PpkA

The function of PpkA has been linked to TagR, as its autophosphorylation is significantly reduced in a Δ*tagR* background^19^. Given our data showing that a TagQ/TagR complex is formed upon membrane damage, we sought to investigate whether there is a direct link between PpkA and the TagQ/TagR complex. For this, we cloned C- and N-terminal FLAG tags on PpkA, generating a PAO1 *ΔretS tssB1-mNG ppkA-FLAG* strain. Live-cell fluorescence microscopy revealed that only the N-terminal tag allowed dueling similar to the parental strain (Fig. S5A). This strain was also equally efficient at killing prey cells as the parental strain (Fig. S2). Using this strain, we purified PpkA directly from *P. aeruginosa* using Co-IP and confirmed purity by western blot and MS analysis (Fig. S5B).

Considering the low abundance of PpkA and the existence of the TagQ/TagR complex only immediately after membrane damage, we attempted an in vitro Co-IP approach to assay complex binding with PpkA. Unfortunately, a variety in yields and overall low stability of the protein produced contradicting results.

To model potential interactions, we used AlphaFold3^29^ to predict structures of TagR complexes with TagQ, the folded periplasmic domain of PpkA, as well as both proteins at once (Figs. 6B and S6). Both the TagR/PpkA as well as the TagR/TagQ/PpkA models contained a 13-residue loop in TagR (residues 261–273) interacting with a corresponding pocket in PpkA (Fig. 6C). Analysis of the TagR sequence using InterProScan^30^ identifies residues 127–359 as homologous to the FGE-sulfatase protein superfamily. To test whether this loop played a role in complex formation, we cloned multiple point mutants, a complete deletion of the loop in TagR, and a replacement of all polar amino acids with alanine (R273A, Q270A, T268A, Q263A, N262A, Y261A). Live-cell imaging of these constructs showed a complete lack of dueling in both the loop deletion and alanine replacement mutants, but no effect from any of the point mutations (Fig. 6E, F). To confirm that both loop deletion (*tagR* Δ261–273) and alanine replacement mutants were expressed and stable, TagR expression levels in the mutants and parental strain were compared by Western blot. This analysis showed that all tested mutants are expressed and stable similarly to the wild-type TagR (Fig. S5C).

In summary, our data suggest a potential interaction between PpkA and the TagQ/TagR complex, mediated through a 13-residue loop in TagR.

## Discussion

The “tit-for-tat” response mechanism in *P. aeruginosa* is known to be essential for its ability to compete with other bacteria in the environment. The membrane damage sensor module consisting of TagQ/TagR/TagS and TagT together with the IM kinase PpkA spans from the outer membrane to the cytoplasm. Here, we show here that TagQ forms a 2:2 complex with TagR. Most importantly, we saw that complex formation is induced by several different types of membrane damage, including PMBN, active exclusively at the OM. Our observation that pretreatment of cells with polymyxin B immediately prior to lysis increased purification yields by 6-fold further supports this conclusion.

Using mass photometry, we were able to show that TagR can form homodimers in the absence of TagQ. However, this occurs only at concentrations significantly higher than those predicted in vivo, suggesting TagQ has a stabilizing role. The TagR dimer appears to be a symmetrical structure, as suggested by our cryo-EM data and AlphaFold3 modeling. Unfortunately, we were unable to reconstruct a 3D model of the TagR dimer or the TagQ/TagR complex, potentially due to a high degree of flexibility in both complexes. Where the interaction with TagQ occurs remains unknown as we were unable to obtain structural information about the entire complex, but our data showing that the interaction persists in the TagQ C30A mutant suggests that the lipidation is not an essential component of the binding interface. While AF3 produces a model of a TagR/TagQ complex, the low-confidence of the prediction at the interface suggests the real binding parameters may be different.

The IM TagS/TagT complex is similar to known ABC transporters and has been suggested by previous models to interact directly with other components of the sensor module or act as a transporter^18^. However, the complex does not appear to be involved in the localization of either TagQ or TagR^18^. Moreover, while we show here that ATPase activity is required in vivo for sensor module function, there do not appear to be any stable interactions that form with known sensor module members. However, we do identify in vivo interactions with known structural components of the T6SS such as TssL and TssB as well as other cellular proteins not related to T6SS function. This suggests that TagS/TagT may play a role in other pathways, transfer energy through transient interactions, or transport small molecules that were not detectable by our approaches.

Phosphorylation of Fha by PpkA has long been understood to be essential for H1-T6SS assembly in *P. aeruginosa*. The subsequent finding that TagR was involved in PpkA activation suggested that other components of the sensor module likely function by promoting this activity. While we were unable to conclusively detect the formation of a complex, structural modeling followed by live-cell imaging of mutants was able to predict a possible interaction. In this, a TagR dimer, stabilized by two copies of TagQ, binds two copies of the folded periplasmic domain of PpkA, bringing them in close enough proximity to promote dimerization. This is possible due to an intrinsically disordered region between the N and C terminal folded domains of PpkA. The binding is mediated by the FGE domain in TagR, previously reported to be important for protein-protein interactions, including in T6SS related proteins^31^. Indeed, this model explains why a TagQ deletion mutant still shows occasional H1-T6SS activity, as TagR dimers would occasionally form for long enough to engage PpkA even in the absence of TagQ. As such, it is tempting to assume that TagQ, highly abundant and localized to the OM, is the first step in the membrane damage sensor of *P*. *aeruginosa*, forming a stable complex with TagR and allowing the dimerization of PpkA to occur. Our recent finding that OM damage alone is sufficient to trigger a sensor-module-dependent firing response seems to support the role of TagQ in this model^16^.

**Fig. 7:**
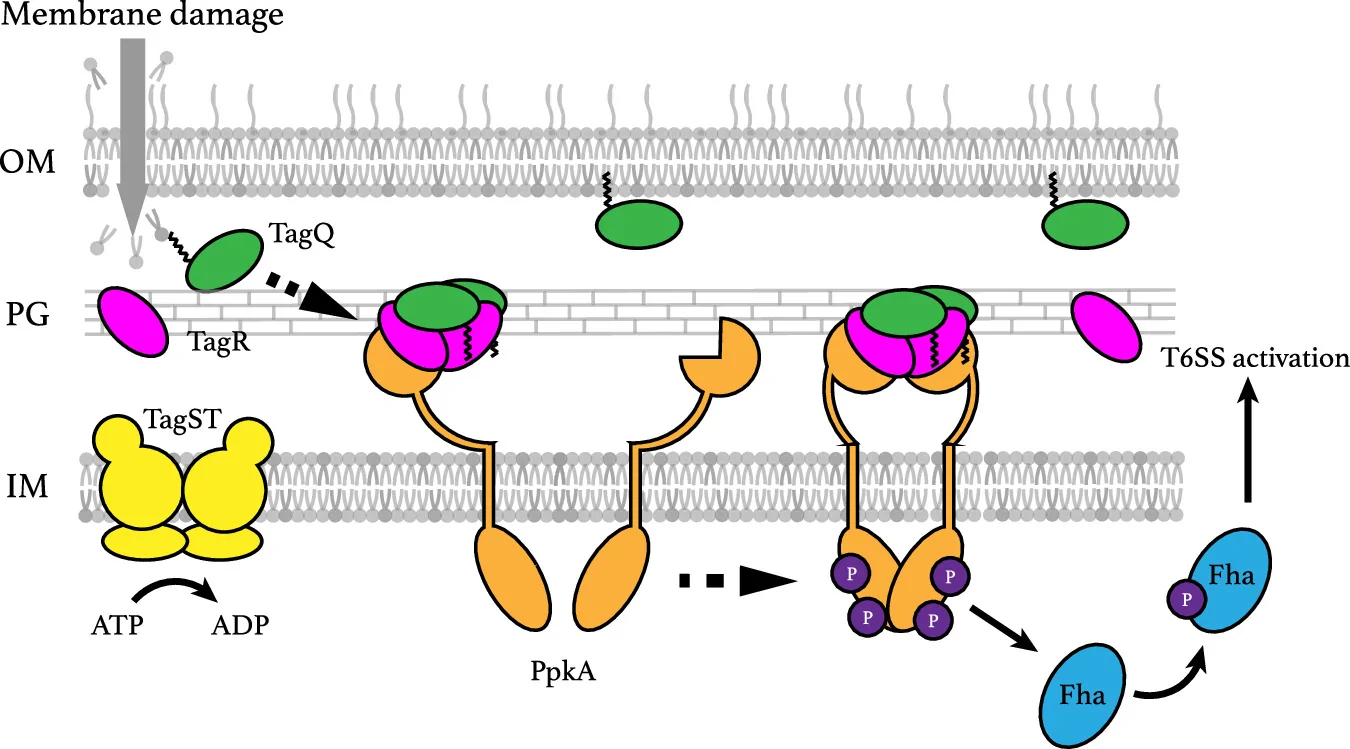
Proposed model of H1-T6SS activation in *P. aeruginosa*. Membrane damage at the OM dislodges TagQ, promoting formation of a stable TagQR complex. This complex in turn binds two copies of PpkA, promoting dimerization, autophosphorylation, and phosphorylation of Fha, initiating T6SS assembly. While TagST ATPase activity is also required, it is not directly bound to any sensor module or TPP component and carries out a yet unknown role.

Taken together, these results allow us to present an updated model of H1-T6SS activation in *P. aeruginosa* (Fig. 7). Damage at the OM promotes the formation of a 2:2 complex between TagQ and TagR, possibly through the release of TagQ from the inner leaflet of the OM itself. This step occurs upstream of PpkA activity and does not require the presence of the lipidation at cysteine 30 of TagQ. Given the low likelihood of TagR existing as a dimer in vivo, TagQ likely binds two copies of TagR sequentially, stabilizing the interaction. The TagS/TagT complex plays an unknown role requiring ATPase activity. The TagQ/TagR complex in turn binds PpkA, possibly promoting dimer formation by binding two copies simultaneously via interactions with symmetrical binding sites in the TagR dimer. This results in the autophosphorylation of PpkA, increasing its activity and subsequent phosphorylation of Fha, promoting H1-T6SS assembly.

Curiously, unlike all known envelope stress responses in bacteria, the activation of PpkA does not recruit or activate proteins responsible for downstream gene regulation^32^. The response to incoming damage in the case of H1-T6SS is required to be immediate to effectively strike the attacking cell, while envelope stress responses usually respond to long-lasting environmental changes^33^. A similar feature is also seen in the Psp envelope stress response, where PspA acts as part of the signaling cascade, sequestering the regulator PspF, but can also itself respond to inner membrane damage by blocking gaps in the inner leaflet and preventing proton leakage^34^. This suggests that H1-T6SS regulation in *P. aeruginosa* might represent a unique response to membrane damage among Gram-negative bacteria.

Functional crosstalk between bacterial secretion systems has previously been reported for most known systems^35^. One limitation of this study is the absence of high-resolution structural data that could confirm the conclusions drawn from biochemical experiments and modeling. While AlphaFold3 is a powerful predictive tool, and in this case largely aligned with our observations, we were unable to determine a sequence-specific mechanistic link between TagQ, TagR, and PpkA. Future work should aim to correct this deficit by obtaining structures of the individual proteins and complexes. Furthermore, follow-up experiments could explore the TagS/TagT complex in greater detail, which we have established has an in vivo interactome that is not exclusive to T6SS components. In summary, our work significantly expands our understanding of T6SS assembly regulation in *P. aeruginosa* and enables further research into the molecular basis of a unique membrane damage response.

## Methods

### Protein purification

For protein purification from *P. aeruginosa*, liquid lysogeny broth (LB) cultures were inoculated from frozen bacterial stock and grown overnight at 37°. The next day, expression cultures were inoculated 1:1000 using overnight cultures, supplemented with 20 μg/ml irgasan/triclosan (Sigma), and grown in LB overnight for a minimum of 12 h at 37 °C. Cells were harvested by 10-min centrifugation at 3000 × *g* and resuspended in lysis buffer. Lysis was performed using a Stansted pressure cell homogenizer (French press) with integrated water cooling using a Lauda microcool MC350 system. Cells were passaged twice at a minimum of 160 MPa. Cell lysate was cleaned using low speed (16,000 × *g* 45 min and high speed (100,000 × *g* 35 min) centrifugation. For membrane proteins, membranes were harvested after cell lysis by cleaning the lysate by low speed (10,000 × *g* 80 min) centrifugation, pelleting the membranes by high speed centrifugation (100,000 × *g* 45 min), and resuspension of membranes using a dounce homogeniser. Lysates were syringe filtered using a 0.45 μm filter. Lysis buffers contained 1% DDM unless stated otherwise. Running buffers contained 0.02% DDM. For purification, lysates were loaded onto anti-DYDYKDDDDK (FLAG) resin (GenScript) by co-incubation with sample at 4 °C. After loading, the bead mixture was loaded onto empty plastic or glass gravity flow columns. Beads were washed with at least 20 column volumes of wash buffer. For elution, beads were incubated at 4 °C with elution buffer equivalent to the bed volume and containing 0.6 mg/ml of DYKDDDDK peptide (GenScript). After this initial elution, three more elutions equivalent to the bed volume and containing 0.1 mg/ml of peptide were performed. Elutions were pooled and either used for analysis or further purified. For further purification using SEC, samples were run on an AKTA pure system using pre-packed columns (Cytiva). For further purification using Strep beads, sample was loaded onto Strep-Tactin XT 4Flow beads (IBA) in a glass or plastic gravity flow column, washed using buffer with at least 10× bed volume, and eluted in elution buffer containing 50 mM biotin. For magnetic bead purifications, Anti-FLAG M2 Magnetic Beads (Sigma) or MagStrep “type3” XT beads (IBA) were incubated with samples for 30 min and washing and elution steps were carried out as described for gravity flow approaches.

For protein purification from *E. coli*, liquid cultures, supplemented with required antibiotics, were inoculated using overnight cultures of target strains and grown to an OD600 of 0.6. Protein expression was induced either via addition of 500 μM of IPTG (AppliChem) or 0.1% Arabinose (Sigma) to the culture. Cultures were incubated overnight at 18 °C. Cells were harvested as above. Cell lysis was performed using sonication (Branson). Cell lysate was cleaned as described. Affinity purification using pre-packed and self-packed Ni-Resin columns (Cytiva, ThermoFisher) was performed on the AKTA start or AKTA pure system. SEC was performed on the AKTA pure system using pre-packed columns (Cytiva). For membrane proteins, running buffers contained 0.02% DDM.

### Bacterial mutagenesis

For the generation of mutants used in this study, deletions, point mutations, and insertions were generated on the chromosome of *P. aeruginosa* PAO1 using the suicide vector pEXG2 as described by ref. ^36^ in combination with *E. coli* strain SM10(λpir). Briefly, a homologous recombination construct was cloned into pEXG2 and transferred to *P. aeruginosa* via conjugation by SM10 ^37^. Positive and negative selection were carried out on LB agar plates, followed by colony PCR and Sanger sequencing (Microsynth) to confirm mutation of target sequence.

### Quantitative killing assay

Performed as described previously^12^. Overnight liquid cultures in 3 ml LB of strains of interest were set up, supplemented with antibiotics when necessary. The following day, 100 μl of ON culture was added to a fresh 3 ml of LB without antibiotics. Incubation temperatures were 37 °C for *P. aeruginosa* PAO1 and 30 °C for *A. baylyi* ADP1 strains. Following growth for 3 h cultures were concentrated to approximately OD_600_ 10. Strains were mixed in a 5:1 PAO1:ADP1 ratio and plated by multichannel on LA plates in triplicate. Dried spots were incubated for 3 h at 37 °C. Culture spots were punched out, transferred to sterile 1.5 ml Eppendorf tubes with 500 μl LB, and mixed vigorously by vortex. Subsequently, a 1:10 serial dilution up to 10^−6^ was plated on LA/Irg20 (PAO1) and LA/Strep100 (ADP1) recovery plates. CFUs were counted the following day.

### Fluorescence live-cell microscopy

The microscope setup used for all image acquisition in this study was described previously in refs. ^7,38,39^. Briefly, overnight cultures of *P. aeruginosa* were streaked on plain LB plates and used to inoculate day cultures the next day on fresh LB plates. Cells were grown for 2 h at 37 °C, were scraped, washed twice with LB, and diluted to an OD600 of 10. 1.2 μl of each sample was spotted on pads consisting of 1% agarose in 2/3 phosphate buffered saline (phosphate buffered saline (PBS)) and 1/3 LB. Samples were incubated for 10 min at 30 °C before imaging. For imaging involving treatments, polymyxin B (20 μg/ml), EDTA (2 mM), PMBN (40 μg/ml), and melittin (40 μg/ml) were added directly to molten pads prior to pad solidification. When measuring treatment effects, samples were not pre-incubated, and only the initial video imaged on the pad was used for analysis. Most images were collected every 3 s for 2 min. Fluorescence channel exposure time was set to 150 ms and brightfield exposure was set to 50 ms. All imaging was repeated for a minimum of three technical replicates consisting of separately incubated cultures measured on the same day.

### Image analysis

All images were initially pre-processed using Fiji software as previously described^12,39,40^. Firing events, i.e., spots, were detected on the GFP channel. For this, the center plane of the stack was background-subtracted using a white top-hat filter from scikit-image.morphology and a radius of *r* = 3px^41^. The image was then normalized using a min-max scaling as follows:

$${I}_{norm}=\frac{{I}-{I}_{min}}{{I}_{max}-{I}_{min}}\cdot 255$$

with *I*_*norm*_ being the normalized image, *I* the image itself, and *I*_*min*_ and *I*_*max*_ the minimal and maximal intensities of the image. Spots were detected by searching for local maxima in intensities in the center plane of the image using Extrema from scikit-image.morphology^41^. The sd parameter was empirically determined from manual annotation of a subset of images, but was sd = 20 in most cases. Further parameters were *n* = 2. Intensity maxima were filtered based on the goodness of a fit with a 2D Gaussian (quality threshold of 0.5). Spots were linked in time using LAPtrack with the following tracking parameters^42,43^: The tracking cost cut-off was 5 px, the maximum number of gaps allowed was 2, and the gap closing cost cut-off was 5 px. Tracks with a length smaller than 5 frames were excluded from further analysis. The number of bacteria for each field of view was calculated using the brightfield image: For this, the center plane of the brightfield channel was segmented using the local Sauvola thresholding from scikit-image.filters with a window size of 25 px^41^. Small holes and noise in the binary mask were smoothened by opening (erosion followed by dilation) using the morphologyEx function from OpenCV and a 3 × 3 px kernel^43^. The distance transform was calculated using distanceTransform from OpenCV and then thresholded to identify separated foreground objects with a 35% cut-off on the distance transform^43^. Individual objects were counted using connectedComponents from OpenCV and scikit-image.measure^41,43^. The firing index was calculated by dividing the number of spots in the GFP channel (i.e., firing events) by the median number of bacteria counted per frame over the course of the movie.

### Western blotting

Protein samples used for western blotting analysis were loaded onto precast Mini-Protean SDS-PAGE gels (Bio-Rad) and run through the gels for 30 min at 250 V. Gels were subsequently transferred to 0.2 μm pore nitrocellulose membrane (Cytiva) using the Trans-Blot Turbo transfer system (Bio-Rad). Following transfer, membranes were incubated for a minimum of 1 h in a 5% concentration of skim milk solution (Merck). Primary antibodies were added directly to the solution and incubated for a minimum of hour temperature. Samples were washed with PBST 3× for 5 min and incubated in fresh skim milk solution. Secondary antibodies (anti-rabbit/anti-goat) (LI-COR, Jackson ImmunoResearch) were added and incubated for 1 h with the sample. Blots were imaged either on a VILBER Fusion, after activation with KPL LumiGLO solution (Seracare), or LI-COR Odyssey imagers.

### Mass photometry analysis of protein samples

Mass photometry of all samples^44^ was performed on the Refeyn Two MP (Refeyn). Samples were diluted to an initial concentration of 50 nM, including a final 10x droplet dilution step onto the sample slide. Samples were measured for 1 min. If required, concentration was adjusted to improve quality parameters. Incubation experiments consisted of repeated measurements of one sample droplet, or pre-incubation of a sample prior to droplet dilution. Experimental design and sample processing were done using the AquireMP software package. Analysis of experimental data and generation of plots were done using the DiscoverMP software package.

### AlphaFold structure prediction

All protein structure predictions for this study were done using AlphaFold 3^29^.

### Cryo-EM sample preparation and data collection

For sample preparation, freshly purified TagR sample at a concentration of 3.4 mg/ml in a buffer containing 50 mM HEPES, 150 mM NaCl, and 2 mM TCEP was applied to grids. For each grid 4 μl of the sample was applied to a Quantifoil R1.2/1.3 holey carbon copper grid (Quantifoil Micro Tools), which was mounted in a Vitrobot (Thermo Fisher Scientific) with temperature and humidity set to 4 °C and 95%, respectively. The grid was blotted with a setting of 3 s and rapidly plunge-frozen in liquid ethane. Data were collected using a Titan Krios (Thermo Fisher Scientific FEI) transmission electron microscope equipped with a K2 Summit direct electron detector (Gatan) using SerialEM (RRID:SCR_017293)^45^ in counting mode. During data collection, the defocus was varied between –1.5 and –3 μm and five exposures were collected per hole. Stacks of frames were collected with a pixel size of 0.82 Å/pixel and a total dose of about 51 electrons/Å^2^.

For the Glacios data collection, TagR at a concentration of 3.2 mg/ml was plunge frozen in the same conditions as above. Data were collected using a Glacios (Thermo Fisher Scientific FEI) transmission electron microscope equipped with a K3 Summit direct electron detector (Gatan) using SerialEM (RRID:SCR_017293)^45^ in counting mode. During data collection, the defocus was varied between –1 and –2.5 μm, and one exposure was collected per hole. Stacks of frames were collected with a pixel size of 0.88 Å/pixel and a total dose of about 44 electrons/Å^2^.

### Cryo-EM data processing

The Titan dataset, consisting of 6336 micrographs, was corrected for beam-induced drift using Patch Motion, and the contrast transfer function (CTF) parameters for each micrograph were determined using Patch CTF in cryoSPARC (RRID:SCR_016501)^46^. After curation of micrographs, we selected 5830 micrographs for further processing. Particles were picked with the template picker function in cryoSPARC, using 2D classes showing structural features as templates. Particles were subjected to reference-free 2D classification and subjected to reference-free 2D classification. To sort out heterogeneity, particles were subjected to Hetero Refinement, followed by non-uniform refinement of the most populated class. Particles were subjected to local and global CTF refinement and further non-uniform refinement, yielding a reconstruction at 3.4 Å.

The Glacios dataset, consisting of 650 micrographs, was corrected for beam-induced drift using PatchMotion, and the CTF parameters for each micrograph were determined using Patch CTF in cryoSPARC (RRID:SCR_016501)^46^. After curation of micrographs, we selected 612 micrographs for further processing. Particles were picked with the blob picker function in cryoSPARC and subjected to reference-free 2D classification. 2D classes showing structural features were used as templates for particle picking using the template picker in cryoSPARC. Iterative 2D classification was used to sort particles. Particles were extracted from micrographs with a box size of 217 px, and an initial homogeneous refinement yielded a reconstruction at 6.15 Å. Particles were subjected to non-uniform refinement, followed by 3D classification and subsequent non-uniform refinements, yielding a reconstruction at 4.32 Å.

### Sample preparation for MS-based proteome analysis

#### Cell lysates

*P. aeruginosa* samples (PAO1) were lysed in 80 μl lysis buffer (2 M guanidinium HCl (Gua-HCl)/0.1 M Ammoniumbicarbonate, 5 mM TCEP, phosphatase inhibitor cocktail, Roche) and heated to 95 °C for 10 min. Samples were further sonicated using a BioRuptor (Diagenode, 4 °C, 20 cycles, 30 s on/30 s off). Carbamidomethylation was performed by addition of Chloroacetamide (Sigma, C0267-100G, final concentration 15 mM). Before addition of Trypsin (Promega, 1:50 wt/wt), GuaHCl was diluted 5x by addition of 320 μl ammonium bicarbonate buffer. Trypsin digestion was performed at 37 °C overnight. Samples were acidified by addition of 50 μl 20% TFA solution and purified using BioPure MacroSpin C18 columns (NEST group) according to the protocol of the manufacturer. Purified peptide eluate was dried in a vacuum concentrator and stored at −20 °C until further use.

#### Affinity purifications

Affinity purification eluates were carbamidomethylated by addition of iodoacetamide (20 mM final) at 25 °C in the dark for 30 min. Samples were adjusted to 90 μl final volume (final buffer containing 5% SDS / 10 mM TCEP / 100 mM TEAB). Proteins were purified and digested with the SP3 approach (PMID: 30464214) using a Freedom Evo 100 liquid handling platform (Tecan Group Ltd., Männedorf, Switzerland). In brief, Speed Beads^TM^ (#45152105050250 and #65152105050250, GE Healthcare) were mixed 1:1, rinsed with water, and diluted to the 8 μg/µL stock solution. Samples were adjusted to the final volume of 90 µL and 10 µL of the bead stock solution was added to them. Proteins were bound to the beads by addition of 100 µL of 100% acetonitrile to the samples, which were then incubated for 8 min at RT with a gentle agitation (50 g). After, samples were placed on a magnetic rack and incubated for 5 min. Supernatants were removed and discarded. The beads were washed twice with 160 µL of 70 % (v/v) ethanol and once with 160 of 100% acetonitrile. Samples were placed off the magnetic rack, and 50 µL of digestion mix (10 ng/µL of trypsin in 50 mM triethylammonium bicarbonate) was added to them. Digestion was allowed to proceed at 37 °C for 12 h. After digestion, samples were placed back on the magnet and incubated for 5 min. Supernatants containing peptides were collected and dried under vacuum. Samples were stored at −20 C until further use.

#### Purified proteins

A total of 5 μg purified protein per sample was heated to 95 °C for 10 min and sonicated using a BioRuptor (Diagenode, at 4 C, 10 cycles at 30 s on and 30 s off). Carbamidomethylation was performed by addition of Chloroacetamide (Sigma, C0267-100G, final concentration 15 mM). Samples were digested by incubation with 0.25 μg Trypsin (Promega, V5113) at 37 °C overnight. Tryptic activity was stopped by addition of 5% TFA, and peptides were washed and purified using iST cartridges (Preomics.com) according to the protocol of the manufacturer. Peptide samples were dried by vacuum concentration and stored at −20 °C until further use. All samples were dissolved in LC-MS/MS LC-buffer containing 0.1% FA/in water prior to injection into the mass spectrometer.

### Mass spectrometry analysis

#### QEplus (affinity purifications)

About 0.25 μg of peptides were subjected to LC–MS/MS analysis using a Q Exactive Plus Mass Spectrometer fitted with an EASY-nLC 1000 (both Thermo Fisher Scientific) and a custom-made column heater set to 60 °C. Peptides were resolved using a RP-HPLC column (75 μm × 30 cm) packed in-house with C18 resin (ReproSil-Pur C18–AQ, 1.9 μm resin; Dr. Maisch GmbH) at a flow rate of 0.2 μL min^−1^. The following gradient was used for peptide separation: from 5% B to 10% B over 5 min, to 35% B over 45 min, to 50% B over 10 min, to 95% B over 2 min followed by 18 min at 95% B. Buffer A was composed of 0.1% formic acid in water. Buffer B was composed of 80% acetonitrile and 0.1% formic acid in water. The mass spectrometer was operated in DDA mode with a total cycle time of approximately 1 s. Each MS1 scan was followed by high-collision-dissociation (HCD) of the 10 most abundant precursor ions, with dynamic exclusion set to 45 s. For MS1, 3e6 ions were accumulated in the Orbitrap over a maximum time of 100 ms and scanned at a resolution of 70,000 FWHM (at 200 *m*/*z*). MS2 scans were acquired at a target setting of 1e5 ions, maximum accumulation time of 100 ms and a resolution of 35,000 FWHM (at 200 *m*/*z*). Singly charged ions and ions with unassigned charge state were excluded from triggering MS2 events. The normalized collision energy was set to 27%, the mass isolation window was set to 1.4 *m*/*z* and one microscan was acquired for each spectrum.

#### Orbitrap Lumos (cell lysate)

About 0.25 μg of peptides were resuspended in 0.1% aqueous formic acid and subjected to LC–MS/MS analysis using an Orbitrap Fusion Lumos Mass Spectrometer fitted with an EASY-nLC 1200 (both Thermo Fisher Scientific) and a custom-made column heater set to 60 °C. Peptides were resolved using a RP-HPLC column (75 μm × 36 cm) packed in-house with C18 resin (ReproSil-Pur C18–AQ, 1.9 μm resin; Dr. Maisch GmbH) at a flow rate of 0.2 μL min^−1^. The following gradient was used for peptide separation: from 5% B to 12% B over 5 min to 35% B over 40 min to 50% B over 15 min to 95% B over 2 min followed by 18 min at 95% B. Buffer A was 0.1% formic acid in water and buffer B was 80% acetonitrile, 0.1% formic acid in water. The Orbitrap Lumos mass spectrometer was operated in DDA mode. MS1 scans were acquired in the Orbitrap in profile mode at a resolution of 240,000 FWHM (at 200 *m*/*z*), a scan range from 375 to 1600 *m*/*z*, normalized AGC target set to 250%, and maximum ion injection time mode set to 50 ms. MS2 scans were acquired in the ion trap, normalized AGC target set to 100% and a maximum injection time of 35 ms. Peptides were fragmented by HCD (Higher-energy collisional dissociation) with collision energy set to 35%, and one microscan was acquired for each spectrum.

#### QEHF and Orbitrap Exploris 480 (protein identification)

About 0.025 μg of peptides were subjected to LC–MS/MS analysis using a Q-Exactive HF or Orbitrap Exploris 480 mass spectrometer. QEHF was fitted with an Ultimate 3000 nano-LC (both Thermo Fisher Scientific) and a custom-made column heater set to 60 °C. Peptides were resolved using a RP-HPLC column (75 μm × 30 cm) packed in-house with C18 resin (ReproSil-Pur C18–AQ, 1.9 μm resin; Dr. Maisch GmbH) at a flow rate of 0.3 μL min^−1^. Orbitrap Exploris 480 was fitted with a nanoelectrospray ion source (both Thermo Fisher Scientific). Peptide separation was carried out using an Evosep One chromatographic system (Evosep.com) equipped with a Performance column (150 μm × 15 cm, 1.5 μm, EV1137) and a custom‑made column heater (50 °C) at 500 nl/min flow rate (30 SPD method).

For QEHF analysis, the following gradient was used for peptide separation: from 2% B to 7% B over 5 min to 30% B over 45 min to 50% B over 10 min to 95% B over 2 min followed by 10 min at 95% B. Buffer A was 0.1% formic acid in water, and buffer B was 80% acetonitrile, 0.1% formic acid in water. The QEHF mass spectrometer was operated in DDA mode. MS1 scans were acquired in the Orbitrap in profile mode at a resolution of 120,000 FWHM (at 200 *m*/*z*), a scan range from 350 to 1600 *m*/*z* and maximum ion injection time mode set to 100 ms. MS2 scans were acquired in the Orbitrap, resolution 30,000 FWHM, the AGC target set to 1E5, and a maximum injection time of 50 ms. Peptides were fragmented by HCD (Higher-energy collisional dissociation) with collision energy set to 28%, and one microscan was acquired for each spectrum.

The Orbitrap Exploris 480 mass spectrometer was operated in data dependent acquisition (DDA) mode. MS1 scans were acquired in the Orbitrap in profile mode at a resolution of 120,000 FWHM (at 200 *m*/*z*), a scan range from 350 to 1600 m/z and maximum ion injection time mode set to “auto”. MS2 scans were acquired in the Orbitrap with the AGC target set to “standard”, resolution of 15,000 FWHM, and a maximum injection time mode of “auto”. Peptides were fragmented by HCD (Higher-energy collisional dissociation) with collision energy set to 27%, and one microscan was acquired for each spectrum.

### Protein identification and quantification

#### Immunopurification samples

For affinity pulldown analysis and protein identification samples, the acquired raw files were searched by FragPipe using MSFragger^47^ against the *Pseudomonas aeruginosa* PAO1 (UP000002438, downloaded from UniProt 20220107) and 392 commonly observed contaminants. Default settings were used for the search, search criteria were set as follows: full tryptic specificity was required (cleavage after lysine or arginine residues unless followed by proline), 2 missed cleavages were allowed, carbamidomethylation (C) was set as fixed modification, and oxidation (M) and N-terminal acetylation were set as a variable modification. A target-decoy search strategy was used in order to obtain a protein false discovery rate of 1%. Search results were analysed using the Scaffold software environment. Quantitative results were statistically evaluated using the MSstats R package v.4.9.9.^48^.

#### Cell lysate data and TurboID data

The acquired raw files were imported into the Progenesis QI software (v2.0, Nonlinear Dynamics Limited), which was used to extract peptide precursor ion intensities across all samples applying the default parameters. The generated mgf-file was searched using MASCOT against the UniProt *Pseudomonas aeruginosa* PAO1 database (UP000002438, downloaded from Uniprot 20220107) and 392 commonly observed contaminants using the following search criteria: full tryptic specificity was required (cleavage after lysine or arginine residues, unless followed by proline); 3 missed cleavages were allowed; carbamidomethylation (C) was set as fixed modification; oxidation (M) was applied as variable modifications; mass tolerance of 10 ppm (precursor) and 0.02 Da (fragments) (for QEHF) /0.6 Da (fragments, Orbitrap Lumos ion trap). The database search results were filtered using the ion score to set the false discovery rate (FDR) to 1% at the peptide and protein level, respectively, based on the number of reverse protein sequence hits in the datasets. Quantitative analysis results from label-free quantification were processed using the SafeQuant R package v.2.3.4^49^ to obtain peptide relative abundances. This analysis included global data normalization by equalizing the total peak/reporter areas across all LC-MS runs, data imputation using the knn algorithm, summation of peak areas per protein and LC-MS/MS run, followed by calculation of peptide abundance ratios. Only isoform-specific peptide ion signals were considered for quantification. To meet additional assumptions (normality and homoscedasticity) underlying the use of linear regression models and *t*-tests, MS-intensity signals were transformed from the linear to the log-scale. The summarized peptide expression values were used for statistical testing of between condition differentially abundant peptides. Here, empirical Bayes moderated *t*-tests were applied, as implemented in the R/Bioconductor limma package (http://bioconductor.org/packages/release/bioc/html/limma.html). The resulting per-protein and condition comparison *p*-values were adjusted for multiple testing using the Benjamini-Hochberg method.

### Statistics and reproducibility

No statistical method was used to predetermine sample size. No data were excluded from the analyses; The experiments were not randomized; The Investigators were not blinded to allocation during experiments and outcome assessment.

### Oligonucleotides and plasmids

A summary of all oligonucleotides and plasmids used in this study has been provided as supplementary documents as Supplementary datasets 8 and 9.

### Reporting summary

Further information on research design is available in the Nature Portfolio Reporting Summary linked to this article.

## Supplementary information

Supplementary Information

Description of Additional Supplementary Files

Supplementary Dataset 1

Supplementary dataset 2

Supplementary dataset 3

Supplementary dataset 4

Supplementary dataset 5

Supplementary dataset 6

Supplementary dataset 7

Supplementary dataset 8

Supplementary dataset 9

Supplementary dataset 10

Video 1

Video 2

Video 3

Reporting summary

Transparent Peer Review file

## Source data

Source Data

## Data Availability

Source data are provided with this paper. The MS and proteomics data generated in this study have been deposited in the PRIDE/MassIVE database under PRIDE identifier: PXD059042/MassIVE identifier: MSV000096700 [http://massive.ucsd.edu/ProteoSAFe/status.jsp?task=37a99c6762714a65b09cb18a4343a170]. The Cryo-EM data generated in this study have been deposited in the Electron Microscopy Public Image Archive under identifier EMPIAR-13668 (https://www.ebi.ac.uk/empiar/EMPIAR-13668/) with the title “Raw micrographs of TagQR collected on a Titan Krios G1 with a Gatan K2 Summit detector”. The movies used in this study are available under restricted access due to their large size, access can be obtained by contacting the corresponding author. The AlphaFold models generated in this study are provided as Supplementary dataset 10. Source data are provided with this paper.

## Code availability

Custom code for analysis of the imaging in this work has been provided in the supplementary information as Supplementary datasets 1–6.
