# Supplementary material for: Membrane damage induces TagQ/TagR complex-mediated H1-T6SS assembly in Pseudomonas aeruginosa
Source: Nat Commun. 2026 Sep 28;17:11187. doi: 10.1038/s41467-026-78112-9 (PMC13713981; doi:10.1038/s41467-026-78112-9)
Supplement: Supplementary file 1 [file 41467_2026_78112_MOESM16_ESM.pdf]

nature portfolio

Corresponding author(s): Basler
Last updated by author(s): Apr 1, 2026

# Reporting Summary

Nature Portfolio wishes to improve the reproducibility of the work that we publish. This form provides structure for consistency and transparency in reporting. For further information on Nature Portfolio policies, see our Editorial Policies and the Editorial Policy Checklist.

## Statistics

For all statistical analyses, confirm that the following items are present in the figure legend, table legend, main text, or Methods section.

| n/a | Confirmed | |
|---|---|---|
| ☐ | ☒ | The exact sample size (*n*) for each experimental group/condition, given as a discrete number and unit of measurement |
| ☐ | ☒ | A statement on whether measurements were taken from distinct samples or whether the same sample was measured repeatedly |
| ☐ | ☒ | The statistical test(s) used AND whether they are one- or two-sided<br>*Only common tests should be described solely by name; describe more complex techniques in the Methods section.* |
| ☐ | ☒ | A description of all covariates tested |
| ☐ | ☒ | A description of any assumptions or corrections, such as tests of normality and adjustment for multiple comparisons |
| ☐ | ☒ | A full description of the statistical parameters including central tendency (e.g. means) or other basic estimates (e.g. regression coefficient) AND variation (e.g. standard deviation) or associated estimates of uncertainty (e.g. confidence intervals) |
| ☐ | ☒ | For null hypothesis testing, the test statistic (e.g. *F*, *t*, *r*) with confidence intervals, effect sizes, degrees of freedom and *P* value noted<br>*Give P values as exact values whenever suitable.* |
| ☒ | ☐ | For Bayesian analysis, information on the choice of priors and Markov chain Monte Carlo settings |
| ☐ | ☒ | For hierarchical and complex designs, identification of the appropriate level for tests and full reporting of outcomes |
| ☒ | ☐ | Estimates of effect sizes (e.g. Cohen's *d*, Pearson's *r*), indicating how they were calculated |

*Our web collection on statistics for biologists contains articles on many of the points above.*

## Software and code

Policy information about availability of computer code

| | |
|---|---|
| Data collection | AlphaFold2 - https://github.com/google-deepmind/alphafold<br>AlphaFold3 - https://alphafoldserver.com/<br>AcquireMP version 2.2 |
| Data analysis | cryoSPARC<br>DiscoverMP version 2.2<br>MSFragger doi: https://doi.org/10.1038/nmeth.4256<br>MSstats R package v.4.9.9. (https://doi.org/10.1093/bioinformatics/btu305) (PMID: 37018319).<br>SafeQuant R package v.2.3.4 (PMID:27345528, https://github.com/eahrne/SafeQuant/)<br>R/Bioconductor limma package (http://bioconductor.org/packages/release/bioc/html/limma.html)<br>ImageJ<br>Miniforge (https://github.com/conda-forge/miniforge)<br>Protter (http://wlab.ethz.ch/protter) |

For manuscripts utilizing custom algorithms or software that are central to the research but not yet described in published literature, software must be made available to editors and reviewers. We strongly encourage code deposition in a community repository (e.g. GitHub). See the Nature Portfolio guidelines for submitting code & software for further information.

## Data

Policy information about availability of data

All manuscripts must include a data availability statement. This statement should provide the following information, where applicable:

- Accession codes, unique identifiers, or web links for publicly available datasets
- A description of any restrictions on data availability
- For clinical datasets or third party data, please ensure that the statement adheres to our policy

Source data are provided with this paper. The MS and proteomics data generated in this study have been deposited in the PRIDE/MassIVE database under Pride identifier: PXD059042/MassIVE identifier: MSV000096700 [http://massive.ucsd.edu/ProteoSAFe/status.jsp?task=37a99c6762714a65b09cb18a4343a170]. The movies used in this study are available under restricted access due to their large size, access can be obtained by contacting the corresponding author. The raw Cryo-EM data are available under restricted access due to no map or structure being generated for this study, access can be obtained by contacting the corresponding author. The AlphaFold models generated in this study are provided in the Supplementary Information/Source Data file.

## Research involving human participants, their data, or biological material

Policy information about studies with human participants or human data. See also policy information about sex, gender (identity/presentation), and sexual orientation and race, ethnicity and racism.

| | |
|---|---|
| Reporting on sex and gender | N/A |
| Reporting on race, ethnicity, or other socially relevant groupings | N/A |
| Population characteristics | N/A |
| Recruitment | N/A |
| Ethics oversight | N/A |

Note that full information on the approval of the study protocol must also be provided in the manuscript.

# Field-specific reporting

Please select the one below that is the best fit for your research. If you are not sure, read the appropriate sections before making your selection.

☒ Life sciences ☐ Behavioural & social sciences ☐ Ecological, evolutionary & environmental sciences

For a reference copy of the document with all sections, see nature.com/documents/nr-reporting-summary-flat.pdf

# Life sciences study design

All studies must disclose on these points even when the disclosure is negative.

| | |
|---|---|
| Sample size | No sample sizes were predetermined, for microscopy samples a minimum of three movies were collected for statistical analysis. |
| Data exclusions | No data was excluded from analysis |
| Replication | Biological and technical replicates were performed for all experiments. Structural data was compared between two different datasets recorded using samples from biological replicates. Experiments that were not successfully reproduced were excluded from the study. |
| Randomization | Not applicable, samples were not subdivided into experimental groups. |
| Blinding | Not applicable, data analysis pipelines were run on all data without author intervention. |

# Reporting for specific materials, systems and methods

We require information from authors about some types of materials, experimental systems and methods used in many studies. Here, indicate whether each material, system or method listed is relevant to your study. If you are not sure if a list item applies to your research, read the appropriate section before selecting a response.

## Materials & experimental systems

| n/a | Involved in the study |
|---|---|
| ☐ | ☒ Antibodies |
| ☒ | ☐ Eukaryotic cell lines |
| ☒ | ☐ Palaeontology and archaeology |
| ☒ | ☐ Animals and other organisms |
| ☒ | ☐ Clinical data |
| ☒ | ☐ Dual use research of concern |
| ☒ | ☐ Plants |

## Methods

| n/a | Involved in the study |
|---|---|
| ☒ | ☐ ChIP-seq |
| ☒ | ☐ Flow cytometry |
| ☒ | ☐ MRI-based neuroimaging |

# Antibodies

Antibodies used

Anti-DYKDDDDK 1 Affinity Resin 10mL resin - GenScript Cat. No. L00432
ANTI-FLAG(R) M2 MAGNETIC BEADS - Cat. No. M8823 Merck
Strep-Tactin®XT 4Flow® resin - Cat. No. 2-5010-002 IBA
MagStrep® Strep-Tactin® beads - Cat. No. 2-1613-002 IBA
IRDye® 800CW Goat anti-Mouse IgG Secondary Antibody - Cat. No. 926-32210 LICORbio (1:20000 dilution)
IRDye® 800CW Goat anti-Rabbit IgG Secondary Antibody - Cat. No. 926-32211 LICORbio (1:20000 dilution)
IRDye® 680RD Goat anti-Rabbit IgG Secondary Antibody - Cat. No. 926-68071 LICORbio (1:20000 dilution)
IRDye® 680RD Goat anti-Mouse IgG Secondary Antibody - Cat. No. 926-68070 LICORbio (1:20000 dilution)
Monoclonal ANTI-FLAG® M2 antibody produced in mouse - Cat. No. F1804 Merck (1:1000 dilution)
Polyclonal rabbit anti-TagQ - custom order Genscript (1:10000 dilution)
Polyclonal rabbit anti-TagR - custom order Genscript (1:10000 dilution)

Validation

Antibodies validated by respective manufacturer, please see respective manufacturer websites for validation standards.

# Plants

Seed stocks

N/A

Novel plant genotypes

N/A

Authentication

N/A
