# Supplementary material for: Membrane damage induces TagQ/TagR complex-mediated H1-T6SS assembly in Pseudomonas aeruginosa
Source: Nat Commun. 2026 Sep 28;17:11187. doi: 10.1038/s41467-026-78112-9 (PMC13713981; doi:10.1038/s41467-026-78112-9)
Supplement: Supplementary file 2 [file 41467_2026_78112_MOESM17_ESM.pdf]

nature portfolio

Peer Review File

Membrane damage induces TagQ/TagR complex-mediated H1-T6SS assembly in Pseudomonas aeruginosa

Corresponding Author: Dr Marek Basler

This file contains all reviewer reports in order by version, followed by all author rebuttals in order by version.

Version 0:

Reviewer comments:

Reviewer #1

(Remarks to the Author)
Kolesnikov et al., report on a post-transcriptional mechanism underpinning the activation of Pseudomonas aeruginosa type VI secretion system (H1-T6SS). The authors in particular tried to elucidate the mechanism of action of four proteins, naimly TagQ, TagR, TagS and TagT, in signal transmission from a bacterial membrane damage to phosphorylation of the H1-T6SS component, Fha1. The role of Tag proteins has been previously adressed in several manuscripts. It is known that an eukaryotic-like threonine kinase/phosphatase pair (PpkA-PppA) works with a so called “signal-transmission module” composed of TagQ-TagR-TagS-TagT proteins. This Tag module is conserved in “defensive” T6SS and acts in response to external attacks (bacterial T6SS, antibiotic polymixin B, conjugation). The subcellular localization of Tag proteins has been also previously defined, and they are all found required for proper function of H1-T6SS and initiation of the baseplate assembly (Mougous et al., 2007, Hsu et al., 2009, Basler et al., 2013, Casabona et al., 2013, Liebl et al., 2019).
The work presented here combines microbiology and biochemistry, with some aspects of structural biology. The given title is somehow misleading as there is not evidence on H1-T6SS assembly per se. The authors mostly concentrate to proove the interactions between Tag proteins which seems to be difficult to tacke. Indeed, there is little convincing evidence for Tag interactions in vivo, although the authors present some data on TagQ/TagR complex formation et interaction between TagR and PpkA.

Major comments
- In Figures 1, 4 and 5, only parts of western blots are presented. There is no loading controles and some Figures are not correctly annotated (ex. 6B and S4B).
- For example, for the Figure 4C, normally we would need to have the similar input in the co-IP to be able to conclude whether there is increase in the TagR-TagQ complex formation. One possibility would be to have an intenal control, or to stain the gel for total proteins. The R-Q complex formation is thought to be rapid (response to membrane damage), meaning that it would not require de novo synthesis, so total TagQ and TagR protein quantities should be the same in untreated and treated samples.
- In the first experiments, the authors constructed Pa strains expressing tagged TagQ and TagR proteins. TagQ-Strep strain seems to display significantly higher level of firing in resting state compared to the TagR-Flag strain. How this quantify to the non-tagged wild-type ?
- It would be useful for the reviewers and for the readers to have the MS/MS tables available or at least the list of the most significant hits (data for Figures 1C, 4C and G).

- The experiments adressing TagS-TagT complex activity and search for partners seem not to be perfectly set-up. Figure 4 shows that the activity of the ATPase is requered for T6SS-dependent firing. Then, it shows that TagS and TagT co-IP, which is already published and well known that the ATPase activity necessite two subunits (TagST are members of LolCDE family). Then the authors search to demonstrate the interactions between TagST and other Tag proteins using proximity labeling. First, the strain harbouring TagT-TurboID is much less efficent in firing than the wild-type (Figure 4F, G). However, this looks not to be an issue here as TagT seems to be autobiotinylated -one would expect a protein gel showing the autobiotinylated fusion in supp data-.
The major concern and it is suprising that the authors fused the TurboID to TagT subunit, that is IM attached and facing the bacterial cytoplasme. Therefore, from the design of the experiment one could not expect to biotinylate OM attached proteins, such as TagR or TagQ. The statement claiming that “The TagST interactome does not include sensor module componants” (line 190 and on, Result section) should be thus changed.

Minor comments

- TagR forms a dimer. Figure 1F suggest that there is a complex between one molecule of TagQ and two molecules of TagR. The purify of the complex could not be verified as only western blots are shown. Cryo-EM figure presents twice the same kind of images selected from the grids. It would be more informative to present the whole grid and selected zoomed images. There is basicly no information that we can gather from that experiment.
- Figure 6 demonstrated the interaction between TagQR complex and PpkA. The authors could comment on PA5258 and NuoE, identified as putative targets of PpkA. The demonstration that PpkA interacts with TagQR complex could have been more convincing. The presented gel in Fig. 6B is not well annotated, as there are several additional uncharacterized proteins in the last lane. Inputs and washes of pulldown experiments are classical controls and should be includded.
- Fig6C – AlphaFold prediction of interaction between TagR and PpkA –the figure could show the probability scores and zoom-in to identify and clearly show the aminoacids that are involved in putative interaction.

Reviewer #2

(Remarks to the Author)
Overall

The study by Kolesnikov et al. focuses on the molecular to structural deciphering of the protein-protein interaction cascade connecting the sensing of membrane damages to the activation of the H1-T6SS in Pseudomonas aeruginosa. The authors have used an elegant functional in vivo pull down approach directly in P. aeruginosa to highlight the formation of the TagQ-TagR complex. This work represents a unique biochemistry approach performed on proteins purified directly from P. aeruginosa. The authors make a good point at approaching the physiological conditions in all their experiments. The authors directly demonstrate that TagT ATPase activity is required to activate the H1-T6SS.
While this study explores a previously understudied mechanism at the molecular and structural levels, making it globally significant, key results upon which conclusions are drawn are currently weak and would benefit from further refinement.

Major Comments

- The stoichiometry evaluation of the TagQ-TagR complex is weak. The 1Q:2R complex seems to represent only a fraction of the sample (Fig. 1F). Indeed, the authors stated that “SEC analysis of proteins purified using anti-FLAG beads showed a mixed population across a wide size range” (lines 126-127): how is it possible to infer a precise stoichiometry from such type of data? Thus affirming that the TagQ-TagR is assembling a 1:2 complex is overstating the case. Even the use of mass photometry doesn’t help since the major peak is the 61 kDa and not the broad 148 kDa peak, which the authors are focusing on to determine the stoichiometry of the TagQ-TagR complex.

I would recommend the authors to use complementary approaching: SEC-MALS, Native MS or Isothermal titration calorimetry (ITC) using purified proteins.

- Please explain the presence of a slow-migrating band under the TagQ protein.

- The cryo-EM study of the TagR dimer does not bring important information to the biological question and suffer from lack of quality (Figure 3). I would recommend the authors to present raw EM images to actually see the TagR particles. The very blurry 2D class averages do not help to understand the possible architecture of the TagR dimer. Lastly, why focusing on TagR dimer since it does not represent the physiological state of the protein?

- Analysis of the TagT proxisome using TurboID. The authors claim that the TagT-TurboID fusion remain fully functional as compare to the WT protein (lines 205-206). Nevertheless, results from Fig. 4F show a significant difference of the “firing index” between WT and TagT-TID. The direct consequence of this negative effect of the TID tag on TagT is how to trust the list of interacting partners when the TagT bait is less functional than the WT protein? And this could explain why TagT-TID does not “catch” other components of the sensor module.

- Membrane damage induces TagQ-TagR complex assembly ?
The authors nicely demonstrated and increase amount of TagQR complex recovered after induction of H1-T6SS by membrane damage?
BUT, is it just an increase level of expression? The authors should check by qRT-PCR the level of transcripts.
The authors should use these conditions (membrane damage) to collect higher amount of TagR/TagQ complex for structural analysis by cryo-EM.

- The TagR-PpkA interface: the authors have used Alphafold2 to model the structure of the complex and to propose key residues at the interface. It is not explain why they focused on TagR-PpkA interface. In the previous paragraph they identified a TagQ-TagR-PpkA complex, without determining the precise site of interaction. In addition, any in silico model should be presented with all the confidence graphs and scores (piTM, PAE…) to be properly evaluated. I strongly recommend the authors to show these information at least in the supplementary figures.

Although the authors demonstrated the implication of the TagR loop 261-273 in the overall function of the H1-T6SS, they however (1) did not demonstrate its direct implication in the TagR-PpkA interaction and (2) failed to identified key residues of the interaction interface.
I would recommend the authors to:
1) Perform pulldown purification between TagR (del 261-273) and PpkA
2) To analyze more closely the residues pairs between TagR and PpkA in the interaction interface to predict the best interacting residues and to mutate them.

Minor Comments

- Lines 74-75: "TagR activity has been linked to both the other membrane and inner membrane components of the H1-T6SS damage response mechanism". I guess the authors wanted to write the "outer" membrane and inner membrane components.

- Figure 1D: please provide the molecular weight markers on the left side of the SDS gel.

- Figure 1E: the two gel bands are not at the same scale?

- Lines 121-133: when indicating stoichiometry evaluation, such as 2:1 please provide which protein is "2" and which protein is "1".

- Figure S2, panels A & B: please provide the molecular weight markers on the left side of the SDS gel.

- Study of the phosphoproteome dependent on PpkA: while the authors show clearly that Fha1 is the major target of PpkA they however did not comment on the presence of PA5258 and nuoE in their Volcano plot. What are they putative functions? Are they new PpkA targets? Have they any regulatory function in H1-T6SS?

- Figure 4B: Although the observation that PpkA is co-purified by the TagQR complex is interesting, the authors did not comment on the fact that TagQR complex appear to be much more stable when in presence of PpkA. This conclusion could be drawn only of equal amout of material have been loaded in each lane of the gel.
Line 247: "TagQR forms a stable complex with PpkA". This title should be toned down. No specific element (biochemical or biophysics evidence) in the paragraph characterize the TagQR-PpkA complex as stable.

Conclusion

It is clear that this study brings some insights into the TagQ/TagR/TagS/TagT/PpkA cascade leading to the activation of the H1-T6SS in P. aeruginosa. In that sense, it is of significance to the T6SS field. However, I do not think that all this work support the conclusions and claims, especially the proposed model.

Reviewer #3

(Remarks to the Author)
In this article Kolesnikov et al describes interactions of proteins involved in the activation of Pseudomonas aeruginosa H1-T6SS upon membrane damage. The manuscript is very well written and easy to follow, the in vivo work is well executed, but the biochemistry part lacks additional support to constitute a convincing story. I would highlight a few most important drawbacks that lack clarity:

The cryoEM data needs additional support. First, it is redundant to provide nearly identical results from two instruments. Second, it should be explained how sample was purified and why did authors analyze only TagR, while TagR-TagQ seems to co-purify easily from P. aeruginosa. Third, authors should provide the AlphaFold model represented in different orientations to consolidate their results. In fact, the model of TagR with PpkA periplasmic domain, provided Figure 6 c, raised some questions. The TagR part of the model doesn't seem to resemble the density seen in the CryoEM 2D classes. Can authors be sure that these 2D classes really correspond to TagR? How many particles are in these classes? Does this object at least resemble the expected dimensions?
In the discussion authors say that they were unable to obtain structural information about TagQ/R complex, but it's not clear what were the attempts? CryoEM? AlphaFold?

I am not convinced about the interpretation of figure 5C-D. Authors show that upon membrane damage yields of purified TagQR complex increase, and they conclude (line 234) that membrane damage promoted formation of TagQR complex. Authors provide only the tandem purification results, but in fact single co-IP would be more interesting, i.e. do thee co-purify more TagQ when purifying TagR or vice versa. After tandem purification however we only see the final complex. Seeing that, one cannot exclude that this comes from increased synthesis of both proteins, or better solubilization of both proteins.

In figure 6B authors show co-purification of PpkA with TagQR, however in 6C they provide model with only TagR. Additional control of only TagR-PpkA interaction and TagQ-PpkA interaction is needed in 6B. It would also be interesting to see the Alpha Fold model including TagQ. While authors say that the PpkA dimerized upon interaction with TagR dimer, the

interaction actually separates the two units of PpkA. If I understand correct, and I think it should be better explained in the text, they propose that interaction with TagR will bring the two PpkA periplasmic domains close together and promote dimerization of cytoplasmic domains of PpkA. Regarding the TagR dimer, AF model should be colored by monomer to better appreciate contribution of each domain. It is also important in order to understand the potential contribution of TagQ in promoting the dimerization.

In the legends it is not clear which antibodies have been used and in some cases it seems that couple of different antibodies were used for one same blot, such as in Fig. 1D and Fig. 5C. It is also unclear what was used in Figure 6B as we see 3 proteins with several different tags. As for anti-TagR antibody (Figure S4) it seems to recognize a few bands.

Authors say PpkA only phosphorylates the Fha1, but what are the other two highlighted points in Fig.6A named "PA5258" and "nuoE"?

Minor issues:

Line 130 – please also provide theoretical mass of TagR monomer
Line 135, and 249 – if this has been demonstrated previously, please provide references.
Line 264, should refer to Figure 6B? This paragraph could also end with some intermediate conclusion.
Line 276, should provide zone in residues that comprises "loop deletion"
Line 428, I think PpkA is shown in cyan, not green.

Version 1:

Reviewer comments:

Reviewer #2

(Remarks to the Author)
I would like to acknowledge the considerable work and diligent efforts the authors have undertaken to address all three reviewers' comments.

Although the authors have improved this part, my only "regret" thought is that the authors did not really answer and thus experimentally investigate this point:

"The TagR-PpkA interface: Although the authors demonstrated the implication of the TagR loop 261-273 in the overall function of the H1-T6SS, they however (1) did not demonstrate its direct implication in the TagR-PpkA interaction and (2) failed to identified key residues of the interaction interface. I would recommend the authors to: 1) Perform pulldown purification between TagR (del 261-273) and PpkA 2) To analyze more closely the residues pairs between TagR and PpkA in the interaction interface to predict the best interacting residues and to mutate them. "

Overall, I appreciate the quality of the study and its impact in the T6SS field, fully justifying a publication in Nature Communications.

Reviewer #3

(Remarks to the Author)
In the revised version of the manuscript authors have made effort to obtain better quality data on the stoichiometry of The TagQ-TagR complex. They also performed additional proteome analysis to clarify that the formation of the complex upon membrane damage is not linked to increased synthesis or changes in solubility of the TagQ or TagR units, consolidating their model. They have also clarified modelling part and updated some of the modelling attempts using most recent version of AlphaFold. I believe that at this point obtaining better quality data to support this model would be challenging and that the manuscript at its current state provides novel findings sufficiently supporting the proposed model.

Open Access This Peer Review File is licensed under a Creative Commons Attribution 4.0 International License, which permits use, sharing, adaptation, distribution and reproduction in any medium or format, as long as you give appropriate credit to the original author(s) and the source, provide a link to the Creative Commons license, and indicate if changes were made.
In cases where reviewers are anonymous, credit should be given to 'Anonymous Referee' and the source.
The images or other third party material in this Peer Review File are included in the article's Creative Commons license, unless indicated otherwise in a credit line to the material. If material is not included in the article's Creative Commons license and your intended use is not permitted by statutory regulation or exceeds the permitted use, you will need to obtain permission directly from the copyright holder.
To view a copy of this license, visit https://creativecommons.org/licenses/by/4.0/

REVIEWER COMMENTS

Reviewer #1 (Remarks to the Author):

Kolesnikov et al., report on a post-transcriptional mechanism underpinning the activation of Pseudomonas aeruginosa type VI secretion system (H1-T6SS). The authors in particular tried to elucidate the mechanism of action of four proteins, naimly TagQ, TagR, TagS and TagT, in signal transmission from a bacterial membrane damage to phosphorylation of the H1-T6SS component, Fha1. The role of Tag proteins has been previously adressed in several manuscripts. It is known that an eukaryotic-like threonine kinase/phosphatase pair (PpkA-PppA) works with a so called “signal-transmission module” composed of TagQ-TagR-TagS-TagT proteins. This Tag module is conserved in “defensive” T6SS and acts in response to external attacks (bacterial T6SS, antibiotic polymixin B, conjugation). The subcellular localization of Tag proteins has been also previously defined, and they are all found required for proper function of H1-T6SS and initiation of the baseplate assembly (Mougous et al., 2007, Hsu et al., 2009, Basler et al., 2013, Casabona et al., 2013, Liebl et al., 2019).
The work presented here combines microbiology and biochemistry, with some aspects of structural biology. The given title is somehow misleading as there is not evidence on H1-T6SS assembly per se. The authors mostly concentrate to proove the interactions between Tag proteins which seems to be difficult to tacke. Indeed, there is little convincing evidence for Tag interactions in vivo, although the authors present some data on TagQ/TagR complex formation et interaction between TagR and PpkA.

Major comments
- In Figures 1, 4 and 5, only parts of western blots are presented. There is no loading controls and some Figures are not correctly annotated (ex. 6B and S4B).

Thank you for pointing out several issues with our western blots. We agree that all raw data should be made available and have included all unedited images as supplementary data (Fig. S8). We have also addressed the annotation errors by adding weight and lane markers where necessary.
Regarding loading controls, we believe that it would not be correct to include a loading control for a tandem purification as introducing a doubly-tagged construct that could purify along with the complex may interfere with our proteins of interest.

- For example, for the Figure 4C, normally we would need to have the similar input in the co-IP to be able to conclude whether there is increase in the TagR-TagQ complex formation. One possibility would be to have an internal control, or to stain the gel for total proteins. The R-Q complex formation is thought to be rapid (response to membrane damage), meaning that it would not require de novo synthesis, so total TagQ and TagR protein quantities should be the same in untreated and treated samples.

Thank you for describing your concerns. We agree that it would be prudent to show that the increase in TagQ/R complex formation does not occur due to factors other than

membrane damage. For this we have carried out a full proteome comparison of our *P. aeruginosa* cells when treated with PolymyxinB vs a buffer treatment control. This shows that there is no discernable difference in total abundance of either protein, or indeed any other sensor module component (Fig. 5F). Furthermore, to exclude the possibility that PolymyxinB itself is responsible for changes in solubility that account for a greater proportion of TagQ/R after purification, we performed a comparison of TagQ/R abundance with PB treatment occurring immediately after cell lysis. If PB increased solubility of either protein, it would be visible in these samples. However, treatment of the lysate with PB did not increase TagQ/R complex abundance (Fig. 5E). We believe these experiments conclusively exclude the possibility that the increase of the TagQ/R complex is not related to induction of membrane damage in live *P. aeruginosa* cells.

- In the first experiments, the authors constructed Pa strains expressing tagged TagQ and TagR proteins. TagQ-Strep strain seems to display significantly higher level of firing in resting state compared to the TagR-Flag strain. How this quantify to the non-tagged wild-type ?

We agree that it would be beneficial to show that resting state firing rate does not affect the function of the sensor module and response to membrane damage. To show this we have performed a killing assay to show T6SS-dependent killing of *A. baylyi* ADP1 cells by our *P. aeruginosa* strains. This showed no statistical difference in killing T6SS(+)ve prey when compared to the parental strain (Fig. S2). Additionally, killing is reduced and similar to the parental strain when the prey is T6SS(-)ve. We believe this result confirms that the tested experimental strains retain full function of their T6SS-mediated response to membrane damage, regardless of background T6SS activity.

- It would be useful for the reviewers and for the readers to have the MS/MS tables available or at least the list of the most significant hits (data for Figures 1C, 4C and G).

Thank you for the comment. We have included additional tables of the most significant hits for the listed MS data in the supplementary for additional clarity (Fig. S7).

- The experiments adressing TagS-TagT complex activity and search for partners seem not to be perfectly set-up. Figure 4 shows that the activity of the ATPase is requered for T6SS-dependent firing. Then, it shows that TagS and TagT co-IP, which is already published and well known that the ATPase activity necessite two subunits (TagST are members of LolCDE family). Then the authors search to demonstrate the interactions between TagST and other Tag proteins using proximity labeling. First, the strain harbouring TagT-TurboID is much less efficent in firing than the wild-type (Figure 4F, G). However, this looks not to be an issue here as TagT seems to be autobiotinylated -one would expect a protein gel showing the autobiotinylated fusion in supp data-.
The major concern and it is suprising that the authors fused the TurboID to TagT subunit, that is IM attached and facing the bacterial cytoplasme. Therefore, from the design of the experiment one could not expect to biotinylate OM attached

proteins, such as TagR or TagQ. The statement claiming that "The TagST interactome does not include sensor module componants" (line 190 and on, Result section) should be thus changed.

Thank you for your comments. We agree that it is prudent to address the discrepancy in background firing rates between our experimental strains and have performed a killing assay as detailed in the response to a previous comment (Fig. S2). For the biotinylated TagT, we believe a western blot to be redundant when MS data is available. Regarding the experimental design, it would have been impossible to use the TurboID system in the periplasm as the biotinylation reaction requires ATP which is not available in the periplasm. Our objective with using the system in the cytoplasm was primarily aimed at addressing previous suggestions that TagST acts as a transporter of TagQ, TagR or other proteins as it is indeed similar to the LolCDE complex. These transported proteins would then have been picked up in MS analysis.
In summary we believe that using both a standard pulldown approach as well as the TurboID system allowed us to address a number of hypotheses regarding TagST function and act as complementary experiments. However, we agree with the reviewer that our statement was too broad and have adjusted it accordingly to better reflect our experimental data (Line 201).

Minor comments

- TagR forms a dimer. Figure 1F suggest that there is a complex between one molecule of TagQ and two molecules of TagR.

Thank you for the comment. To address issues with determining the stoichiometry of the complex we have performed additional mass photometry experiments using an experimental setup that allows us to capture the complex at an earlier stage of dissociation (Fig. 1F). This higher quality data has led us to change adjust our conclusion and propose a 2:2 stoichiometry of the complex (Lines 135-141).

The purify of the complex could not be verified as only western blots are shown.

Thank you for your comment. Figure 1E(i) is in fact an SDS-PAGE gel as noted in the figure legend. We have added an additional gel (Fig. S1) showing several prior purification steps as a supplementary figure for further clarity.

Cryo-EM figure presents twice the same kind of images selected from the grids. It would be more informative to present the whole grid and selected zoomed images. There is basicly no information that we can gather from that experiment.

We thank the reviewer for their concern. In response to this and comments from other reviewers we have revised our Cryo-EM section (Lines 175 – 183, Fig. 3). We believe that although we were unable to obtain a high-resolution map of either TagR or the TagQ/TagR complex, the symmetry and size of the TagR particles (Fig. 3) supports our mass photometry data and AF3 modeling in the conclusion that TagR exists as a dimer

at high concentrations. Similarly, the difficulty in converging data is likely due to the high flexibility of TagR and TagQ/TagR, which we believe to be an important observation considering the sensor module operates as a cascade based on protein-protein interactions.

- Figure 6 demonstrated the interaction between TagQR complex and PpkA. The authors could comment on PA5258 and NuoE, identified as putative targets of PpkA.

We thank the reviewer for the question. As both PA5258 and NuoE were considered insignificant hits due to a low Mascot score (confidence of identification) we had omitted them from our results discussion. We attempted to clarify this by marking significant hits with red dots instead of black. To avoid further confusion, we have omitted the labels entirely.

The demonstration that PpkA interacts with TagQR complex could have been more convincing. The presented gel in Fig. 6B is not well annotated, as there are several additional uncharacterized proteins in the last lane. Inputs and washes of pulldown experiments are classical controls and should be includded.

We agree that the interaction with PpkA is not sufficiently characterized, following several attempts to obtain better quality results, we have decided to remove this data from the manuscript as PpkA abundance and stability was of insufficient quality to get consistent results. Instead, we have included additional modeling of potential interactions with PpkA. As the focus of our manuscript is the formation of a TagQ/R complex in response to membrane damage, we do not believe this negatively impacts the major conclusions we present.

- Fig6C – AlphaFold prediction of interaction between TagR and PpkA –the figure could show the probability scores and zoom-in to identify and clearly show the aminoacids that are involved in putative interaction.

We agree that probability scores should be available and apologize for the oversight on our part. Additionally, we have included zoomed-in views of the relevant amino acids in the supplementary data (Fig. 6 and S6).

Reviewer #2 (Remarks to the Author):

Overall

The study by Kolesnikov et al. focuses on the molecular to structural deciphering of the protein-protein interaction cascade connecting the sensing of membrane damages to the activation of the H1-T6SS in Pseudomonas aeruginosa. The authors have used an elegant functional in vivo pull down approach directly in P. aeruginosa to highlight the formation of the TagQ-TagR complex. This work represents a unique biochemistry

approach performed on proteins purified directly from P. aeruginosa. The authors make a good point at approaching the physiological conditions in all their experiments. The authors directly demonstrate that TagT ATPase activity is required to activate the H1-T6SS.

While this study explores a previously understudied mechanism at the molecular and structural levels, making it globally significant, key results upon which conclusions are drawn are currently weak and would benefit from further refinement.

Major Comments

- The stoichiometry evaluation of the TagQ-TagR complex is weak. The 1Q:2R complex seems to represent only a fraction of the sample (Fig. 1F). Indeed, the authors stated that "SEC analysis of proteins purified using anti-FLAG beads showed a mixed population across a wide size range" (lines 126-127): how is it possible to infer a precise stoichiometry from such type of data? Thus affirming that the TagQ-TagR is assembling a 1:2 complex is overstating the case. Even the use of mass photometry doesn't help since the major peak is the 61 kDa and not the broad 148 kDa peak, which the authors are focusing on to determine the stoichiometry of the TagQ-TagR complex.

I would recommend the authors to use complementary approaching: SEC-MALS, Native MS or Isothermal titration calorimetry (ITC) using purified proteins.

Thank you for the detailed comment and suggestions. We agree that the stoichiometry evaluation is not ideal. As our purifications are very low yield, we are unable to use SEC-MALS, Native MS or ITC for this analysis. Regarding mass photometry, our major issue was the dissociation of the complex in a detergent-free buffer (a requirement of mass photometry), which resulted in the broad 148 kDa peak. Indeed, incubation of the sample for even 1 minute longer would lead to complete degradation (Figure S1). However, for these revised results we were able to take advantage of a microfluidics upgrade to the device that allowed for near instantaneous (millisecond) imaging after mixing the complex with running buffer. This allowed us to repeat these measurements and obtain higher quality data. Figure 1F now shows a mixed population of the TagR monomer (61kDa peak), TagR dimer (107kDa peak) and a third peak at 171 kDa. As these data are of much higher quality and are obtained from far higher count numbers, we feel confident to conclude that the stoichiometry is 2:2 (Lines 135-141).

- Please explain the presence of a slow-migrating band under the TagQ protein.

Thank you for your comment. We agree that an explanation of this band would be prudent and have conducted a mass spectromentry experiment to show that this band is the product of N-terminal degradation of TagQ.

- The cryo-EM study of the TagR dimer does not bring important information to

the biological question and suffer from lack of quality (Figure 3). I would recommend the authors to present raw EM images to actually see the TagR particles. The very blurry 2D class averages do not help to understand the possible architecture of the TagR dimer. Lastly, why focusing on TagR dimer since it does not represent the physiological state of the protein?

We thank the reviewer for their comments. As similar issues were raised by all reviewers, we have revised our Cryo-EM section (Lines 175 – 183, Fig. 3). We have made significant attempts to obtain a structure of the TagQ/TagR complex. However, the flexibility of the complex prevented us from obtaining meaningful 2D or 3D data. As our yields are extremely low and the complex cannot be reconstituted *in vitro,* we were unable to perform common optimization steps such as detergent screening etc. We believe that although we were unable to obtain a high-resolution map of either TagR or the TagQ/TagR complex, the symmetry and size of the TagR particles (Fig. 3) supports our mass photometry data and AF3 modeling in the conclusion that TagR exists as a dimer at high concentrations. Similarly, the difficulty in converging data is likely due to the high flexibility of TagR and TagQ/TagR, which we believe to be an important observation considering the sensor module operates as a cascade based on protein-protein interactions.

- Analysis of the TagT proxisome using TurboID. The authors claim that the TagT-TurboID fusion remain fully functional as compare to the WT protein (lines 205-206). Nevertheless, results from Fig. 4F show a significant difference of the "firing index" between WT and TagT-TID. The direct consequence of this negative effect of the TID tag on TagT is how to trust the list of interacting partners when the TagT bait is less functional than the WT protein? And this could explain why TagT-TID does not "catch" other components of the sensor module.

We thank the reviewer for the comments and suggestions. We completely agree that it is important to examine the discrepancy in base levels of T6SS activity. As such we have performed a killing assay, to show T6SS-dependent killing of ADP1 cells by *P. aeruginosa* (Fig. S2). Using this assay we show that there is no statistically significant difference in killing T6SS(+)ve prey when compared to the parental strain. This confirms that the response to membrane damage remains intact in our experimental strains. Similarly, killing of T6SS(-)ve prey is reduced and statistically the same as for the parental strain, suggesting that differences in base firing rates do not impact T6SS function more broadly. We believe these results confirm that the primary role of the H1-T6SS in responding to membrane damage remains intact in our strains and that therefore our pulldown results reflect the interactome in the parental strain.

- Membrane damage induces TagQ-TagR complex assembly ?
The authors nicely demonstrated and increase amount of TagQR complex recovered after induction of H1-T6SS by membrane damage?
BUT, is it just an increase level of expression? The authors should check by qRT-PCR the level of transcripts.

We thank the reviewer for the comment and agree that it would be appropriate to show that the increase is not caused by other means. In response to this comment and similar comments made by other reviewers, we carried out a full proteome comparison of *P. aeruginosa* cells treated with PolymyxinB in comparison to a control sample treated with buffer only. Our results showed that there was no discernable difference in total abundance of either protein, or indeed any other sensor module component (Fig 5F). Furthermore, to exclude the possibility that PolymyxinB itself is responsible for changes in solubility that account for the increased TagQ/R yield, we performed a comparison of TagQ/R abundance with PB treatment occurring immediately after cell lysis. If PB increased solubility of either protein, it would be visible in these samples. However, treatment of the lysate with PB did not increase TagQ/R complex abundance (Fig. 5E). We believe these experiments conclusively exclude the possibility that the increase of the TagQ/R complex is not related to induction of membrane damage in live *P. aeruginosa* cells.

The authors should use these conditions (membrane damage) to collect higher amount of TagR/TagQ complex for structural analysis by cryo-EM.

We appreciate the reviewer's suggestion. Unfortunately, the cryo-EM data obtained during our studies was already done using these conditions. We believe that further yield increases would not have improved our data as explained in response to a previous comment.

- The TagR-PpkA interface: the authors have used Alphafold2 to model the structure of the complex and to propose key residues at the interface. It is not explain why they focused on TagR-PpkA interface. In the previous paragraph they identified a TagQ-TagR-PpkA complex, without determining the precise site of interaction. In addition, any in silico model should be presented with all the confidence graphs and scores (piTM, PAE…) to be properly evaluated. I strongly recommend the authors to show these information at least in the supplementary figures.
Although the authors demonstrated the implication of the TagR loop 261-273 in the overall function of the H1-T6SS, they however (1) did not demonstrate its direct implication in the TagR-PpkA interaction and (2) failed to identified key residues of the interaction interface.
I would recommend the authors to:
1) Perform pulldown purification between TagR (del 261-273) and PpkA
2) To analyze more closely the residues pairs between TagR and PpkA in the interaction interface to predict the best interacting residues and to mutate them.

We thank the reviewer for their suggestions. We apologize for the oversight of omitting confidence data and have included them where necessary (Fig. 6 and S6). Initial modeling of the complex was performed using the AlphaFold2 model which was unable to model an interaction between TagR and TagQ. However, it was able to produce a

high-confidence model of the TagR-PpkA interaction, leading to further work on this interaction interface. We agree that the data regarding interaction of the TagQR complex with PpkA is insufficient and attempted to obtain more additional western blots. However, due to the low abundance of PpkA in the cell (600 copies) and its low stability following lysis of the cells, we were unable to obtain consistent results. Consequently, we have decided to omit the *in vitro* data with PpkA and instead have used AlphaFold3 to model a potential TagQ/TagR/PpkA complex instead (Fig. 6).

Minor Comments

- Lines 74-75: "TagR activity has been linked to both the other membrane and inner membrane components of the H1-T6SS damage response mechanism". I guess the authors wanted to write the "outer" membrane and inner membrane components.

Indeed, thank you for spotting the error.

- Figure 1D: please provide the molecular weight markers on the left side of the SDS gel.

Markers have been added, we apologize for the oversight.

- Figure 1E: the two gel bands are not at the same scale?

This is correct, the two images represent different views of purified TagQ/R complex. Figure 1E(i) is an SDS-PAGE gel, while Figure 1E(ii) is a western blot. We have changed the figure and added an additional SDS-PAGE gel in the supplementary data for greater clarity (Fig. 1 and S1)

- Lines 121-133: when indicating stoichiometry evaluation, such as 2:1 please provide which protein is "2" and which protein is "1".

Thank you for the comment, we have rewritten the text in light of our new mass photometry results and have taken this into account.

- Figure S2, panels A & B: please provide the molecular weight markers on the left side of the SDS gel.

We apologize for the omission and have added the markers (Fig. S3).

- Study of the phosphoproteome dependent on PpkA: while the authors show clearly that Fha1 is the major target of PpkA they however did not comment on the presence of PA5258 and nuoE in their Volcano plot. What are they putative functions? Are they new PpkA targets? Have they any regulatory function in H1-T6SS?

We thank the reviewer for the question and apologize for the confusion. We had omitted both PA5258 and NuoE from our discussion as they were considered insignificant hits due to a low Mascot score (confidence of identification). We attempted to clarify this by identifying significant hits with red dots instead of black. To avoid further confusion, we have omitted the labels entirely.

- Figure 4B: Although the observation that PpkA is co-purified by the TagQR complex is interesting, the authors did not comment on the fact that TagQR complex appear to be much more stable when in presence of PpkA. This conclusion could be drawn only of equal amout of material have been loaded in each lane of the gel.
Line 247: "TagQR forms a stable complex with PpkA". This title should be toned down. No specific element (biochemical or biophysics evidence) in the paragraph characterize the TagQR-PpkA complex as stable.

Thank you for the comment, we agree that the initial data was insufficient and in light of our difficulties in obtaining additional pulldown data have decided to omit this result from the manuscript. Instead, we have presented used a modeling approach to predict possible interactions between TagQ/TagR and PpkA.

Conclusion

It is clear that this study brings some insights into the TagQ/TagR/TagS/TagT/PpkA cascade leading to the activation of the H1-T6SS in P. aeruginosa. In that sense, it is of significance to the T6SS field. However, I do not think that all this work support the conclusions and claims, especially the proposed model.

Reviewer #3 (Remarks to the Author):

In this article Kolesnikov et al describes interactions of proteins involved in the activation of Pseudomonas aeruginosa H1-T6SS upon membrane damage. The manuscript is very well written and easy to follow, the in vivo work is well executed, but the biochemistry part lacks additional support to constitute a convincing story. I would highlight a few most important drawbacks that lack clarity:

The cryoEM data needs additional support. First, it is redundant to provide nearly identical results from two instruments. Second, it should be explained how sample was purified and why did authors analyze only TagR, while TagR-TagQ seems to co-purify easily from P. aeruginosa. Third, authors should provide the AlphaFold model represented in different orientations to consolidate their results. In fact, the model of TagR with PpkA periplasmic domain, provided Figure 6 c, raised some questions. The TagR part of the model doesn't seem to resemble the

density seen in the CryoEM 2D classes. Can authors be sure that these 2D classes really correspond to TagR? How many particles are in these classes? Does this object at least resemble the expected dimensions?
In the discussion authors say that they were unable to obtain structural information about TagQ/R complex, but it's not clear what were the attempts? CryoEM? AlphaFold?

We thank the reviewer for their comments. As similar issues were raised by all reviewers, we have revised our Cryo-EM section (Lines 175 – 183, Fig. 3). We have both prior to submission and during the revision process made significant attempts to obtain a structure of the TagQ/TagR complex. As we were unable to reconstitute the complex *in vitro* our attempts were CryoEM as well as AlphaFold modeling (Fig. S6). However, the flexibility of the complex prevented us from obtaining meaningful 2D or 3D data. As our yields are quite low and the complex cannot be reconstituted *in vitro,* we were unable to perform common optimization steps such as detergent screening etc. We believe that although we were unable to obtain a high-resolution map of either TagR or the TagQ/TagR complex, the symmetry and size of the TagR particles (Fig. 3) supports our mass photometry data and AF3 modeling in the conclusion that TagR exists as a dimer at high concentrations. From 6336 micrographs, around 750.000 particles were extracted that corresponded to this particle species with no other obvious species observed. We believe this in combination with our SDS-PAGE and MS data confirms the identity of these particles as TagR. The difficulty in converging data is likely due to the high flexibility of TagR and TagQ/TagR, which we believe to be an important observation considering the sensor module operates as a cascade based on protein-protein interactions. We acknowledge that the particles look slightly different from the AF3 modeling (Fig. 6 and S6) but believe that is likely due to the high flexibility of all complex components. This would also explain why our experiments with individual residues in the TagR interaction loop required multiple mutations to have a significant effect. It is also possible that the interactions observed in the AF3 model represent one of the possible conformations.

I am not convinced about the interpretation of figure 5C-D. Authors show that upon membrane damage yields of purified TagQR complex increase, and they conclude (line 234) that membrane damage promoted formation of TagQR complex. Authors provide only the tandem purification results, but in fact single co-IP would be more interesting, i.e. do thee co-purify more TagQ when purifying TagR or vice versa. After tandem purification however we only see the final complex. Seeing that, one cannot exclude that this comes from increased synthesis of both proteins, or better solubilization of both proteins.

We thank the reviewer for the comment and agree that it would be appropriate to show that the increase is not caused by other means. In response to this comment and similar comments made by other reviewers, we carried out a full proteome comparison of *P. aeruginosa* cells treated with PolymyxinB in comparison to a control sample treated with buffer only. Our results showed that there was no discernable difference in total abundance of either protein, or indeed any other sensor module component (Fig. 5F).

Furthermore, to exclude the possibility that PolymyxinB is responsible to the increase in yield due to a solubilization effect, we performed a comparison of TagQ/R abundance with PB treatment occurring immediately after cell lysis. If PB increased solubility of either protein, it would be visible in these samples. However, treatment of the lysate with PB did not increase TagQ/R complex abundance (Fig. 5E). We believe these experiments conclusively exclude the possibility that the increase of the TagQ/R complex is not related to induction of membrane damage in live *P. aeruginosa* cells.

In figure 6B authors show co-purification of PpkA with TagQR, however in 6C they provide model with only TagR. Additional control of only TagR-PpkA interaction and TagQ-PpkA interaction is needed in 6B. It would also be interesting to see the Alpha Fold model including TagQ. While authors say that the PpkA dimerized upon interaction with TagR dimer, the interaction actually separates the two units of PpkA. If I understand correct, and I think it should be better explained in the text, they propose that interaction with TagR will bring the two PpkA periplasmic domains close together and promote dimerization of cytoplasmic domains of PpkA. Regarding the TagR dimer, AF model should be colored by monomer to better appreciate contribution of each domain. It is also important in order to understand the potential contribution of TagQ in promoting the dimerization.

We thank the reviewer for the comments and suggestions. Initial modeling of sensor module interactions was carried out using AF2, which was unable to model an interaction between TagR and TagQ. This was the primary reason for working with the TagR-PpkA model. In light of the availability of AF3, we have added additional modeling data (Fig. 6 and 6S). As the TagQ/TagR model is still relatively low confidence, we have avoided speculation based on it. Furthermore, we have re-written the text to better illustrate our hypothesis regarding PpkA activation via the TagQR complex.

In the legends it is not clear which antibodies have been used and in some cases it seems that couple of different antibodies were used for one same blot, such as in Fig. 1D and Fig. 5C. It is also unclear what was used in Figure 6B as we see 3 proteins with several different tags. As for anti-TagR antibody (Figure S4) it seems to recognize a few bands.

Thank you for the comment. We agree that in some cases the data was presented in a potentially confusing manner and have adjusted all figure legends accordingly.

Authors say PpkA only phosphorylates the Fha1, but what are the other two highlighted points in Fig.6A named “PA5258” and “nuoE”?

We thank the reviewer for pointing this out and apologize for the oversight. Both PA5258 and NuoE were labeled as insignificant hits due to a low Mascot score (confidence of identification) and were thus not discussed. To avoid further confusion, we have omitted the labels entirely.

Minor issues:

Line 130 – please also provide theoretical mass of TagR monomer

Thank you, noted and changed.

Line 135, and 249 – if this has been demonstrated previously, please provide references.

Thank you for the comment, we apologize for the oversight and have added the correct references in the text.

Line 264, should refer to Figure 6B? This paragraph could also end with some intermediate conclusion.

We have re-written the paragraph to better reflect our data.

Line 276, should provide zone in residues that comprises "loop deletion"

Noted and added to text.

Line 428, I think PpkA is shown in cyan, not green.

Noted and changed.

Reviewer #2 (Remarks to the Author):

I would like to acknowledge the considerable work and diligent efforts the authors have undertaken to address all three reviewers' comments.

Although the authors have improved this part, my only “regret” thought is that the authors did not really answer and thus experimentally investigate this point:

“The TagR-PpkA interface: Although the authors demonstrated the implication of the TagR loop 261-273 in the overall function of the H1-T6SS, they however (1) did not demonstrate its direct implication in the TagR-PpkA interaction and (2) failed to identified key residues of the interaction interface. I would recommend the authors to: 1) Perform pulldown purification between TagR (del 261-273) and PpkA 2) To analyze more closely the residues pairs between TagR and PpkA in the interaction interface to predict the best interacting residues and to mutate them. “

Overall, I appreciate the quality of the study and its impact in the T6SS field, fully justifying a publication in Nature Communications.

Response:
We thank the reviewer for their kind words and their helpful and encouraging comments throughout the review process. We hope to address any outstanding questions in a subsequent work.

Reviewer #3 (Remarks to the Author):

In the revised version of the manuscript authors have made effort to obtain better quality data on the stoichiometry of The TagQ-TagR complex. They also performed additional proteome analysis to clarify that the formation of the complex upon membrane damage is not linked to increased synthesis or changes in solubility of the TagQ or TagR units, consolidating their model. They have also clarified modelling part and updated some of the modelling attempts using most recent version of AlphaFold. I believe that at this point obtaining better quality data to support this model would be challenging and that the manuscript at its current state provides novel findings sufficiently supporting the proposed model.

Response:

We thank the reviewer for their support and helpful guidance during the review process.
