# Supplementary material for: Membrane damage induces TagQ/TagR complex-mediated H1-T6SS assembly in Pseudomonas aeruginosa
Source: Nat Commun. 2026 Sep 28;17:11187. doi: 10.1038/s41467-026-78112-9 (PMC13713981; doi:10.1038/s41467-026-78112-9)
Supplement: Supplementary file 3 [file 41467_2026_78112_MOESM1_ESM.pdf]

Supplementary Information

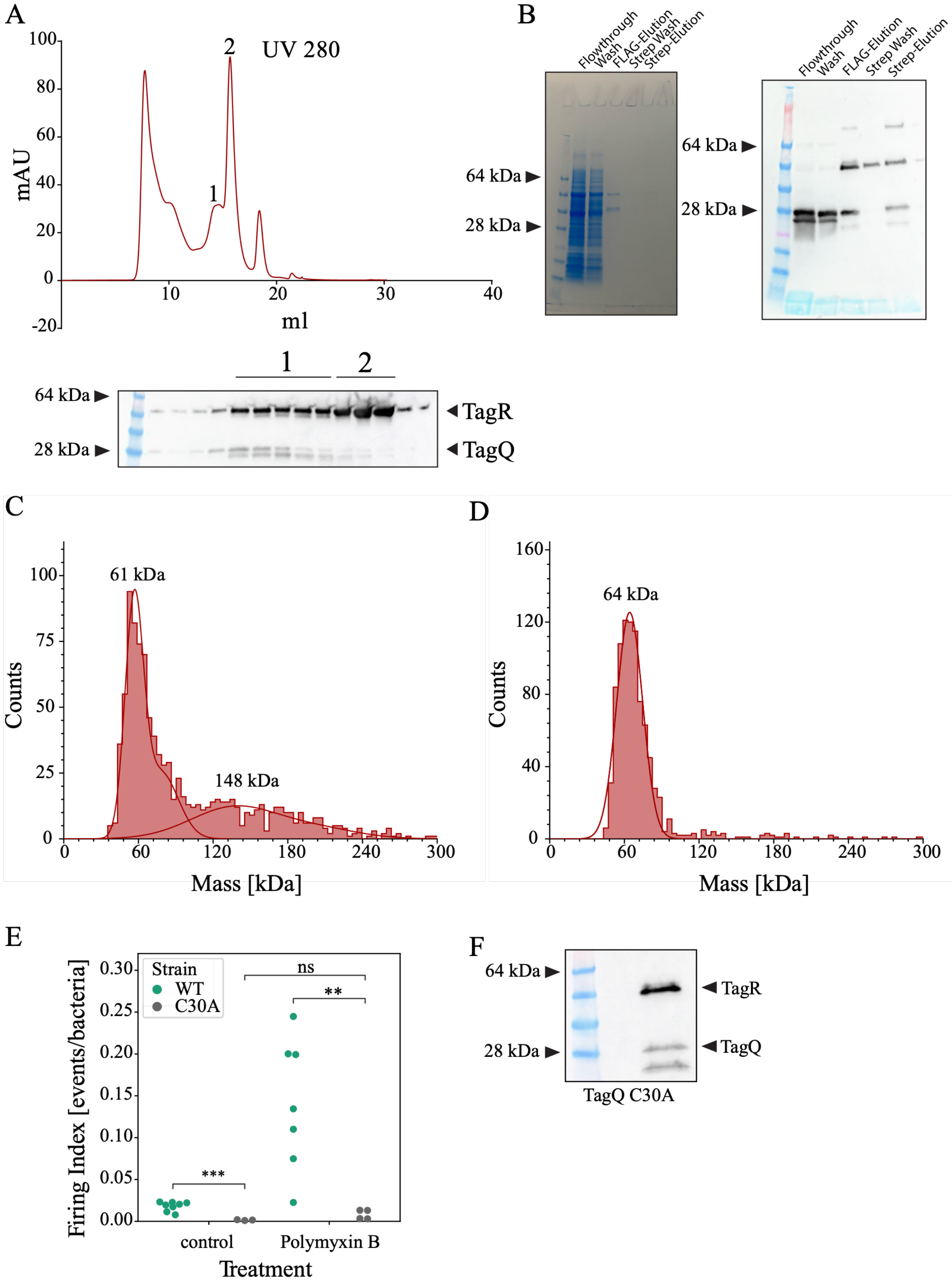

Figure S1: The TagQ/TagR complex is unstable in the absence of detergent, but forms *in vivo* with a C30A TagQ mutant.

(A) UV280 trace of FPLC SEC analysis of proteins purified from *P. aeruginosa* strain with FLAG tag on TagR and western blot of major elution peaks using anti-TagR and anti-TagQ antibodies. (B) SDS-PAGE gel and western blot using anti-TagR and anti-TagQ antibodies showing FLAG and Strep column purification of TagQ/TagR complex. (C) Mass photometry analysis of TagQR purified by tandem affinity showing aggregate counts as histograms with mass estimates derived from fitted normal distributions. Two populations can be identified with estimated masses of 61 kDa and 148 kDa respectively. (D) Incubation of the TagQR sample in a DDM-free buffer for longer than 1 minute results in the complete elimination of the 148 kDa peak. (E) Independent t-test statistical analysis of live cell imaging firing events per bacterium on a single frame for WT and TagQ C30A mutant strains. p-values: $* = p < 0.05$, $** = p < 0.01$, $*** = p < 0.001$, $**** = p < 0.0001$. Two-sided independent student's t-test. All exact p-values available in supplementary table. (F) Western blot using anti-TagQ and anti TagR antibodies of TagQR complex purified by tandem affinity from TagQ C30A *P. aeruginosa* strain.

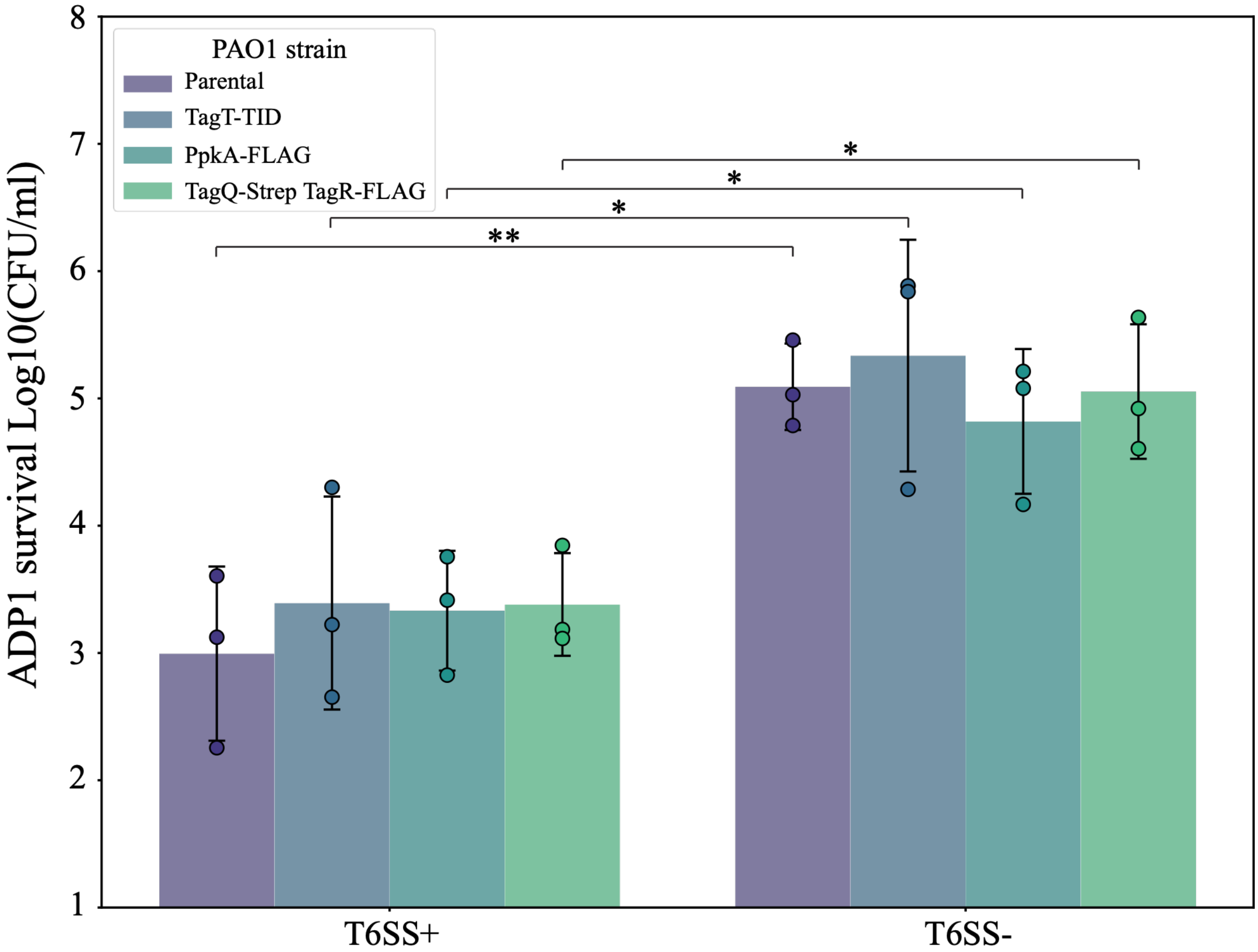

Figure S2: Killing assay shows comparable recovery of prey cells between parental and experimental strains.

Barplot showing recovery of T6SS +ve or T6SS -ve ADP1 cells following incubation with parental or experimental PAO1 strains. Statistical significance measured using standard ANOVA test. p-values: $* = p < 0.05$, $** = p < 0.01$, $*** = p < 0.001$, $**** = p < 0.0001$.

A

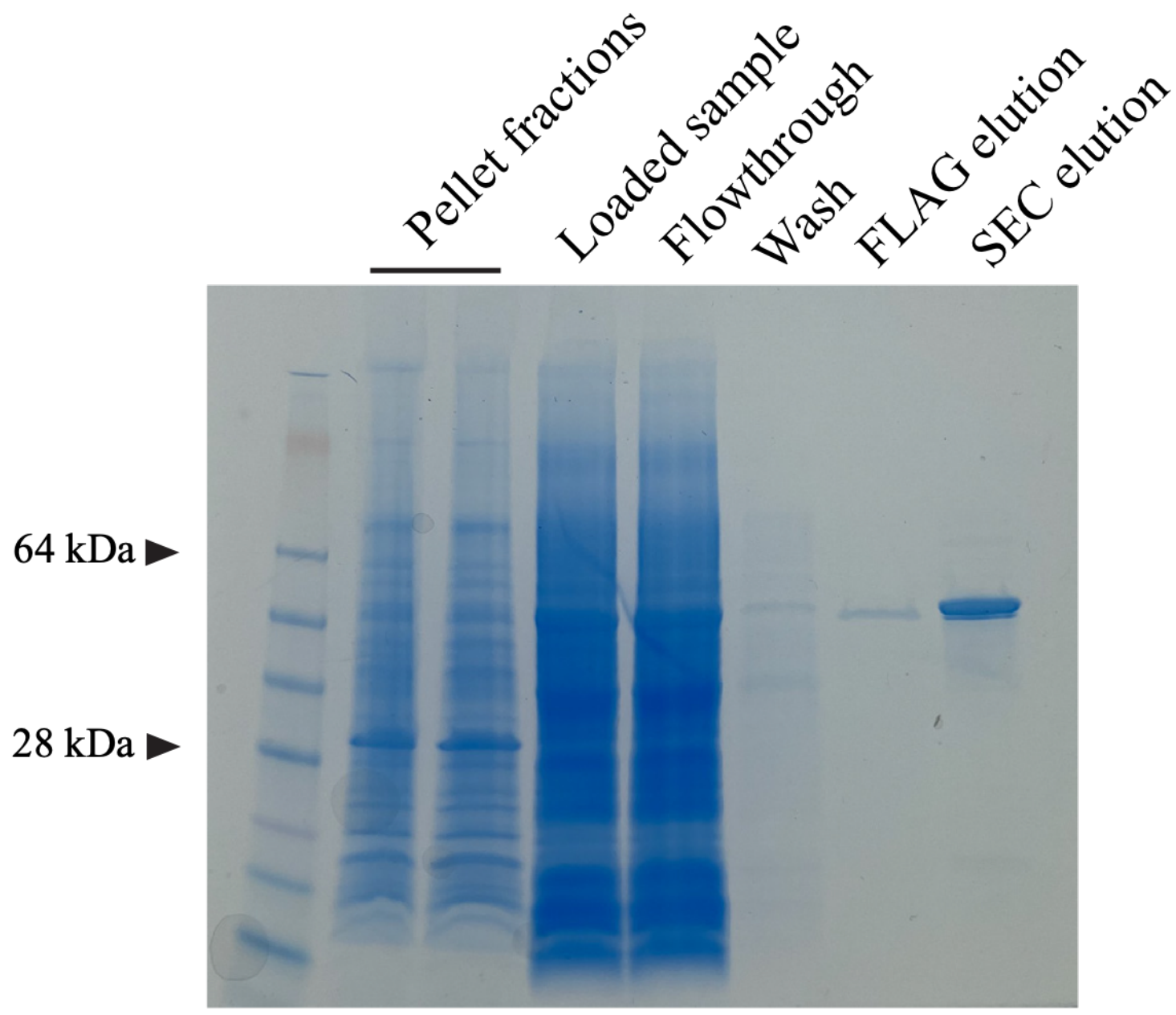

B

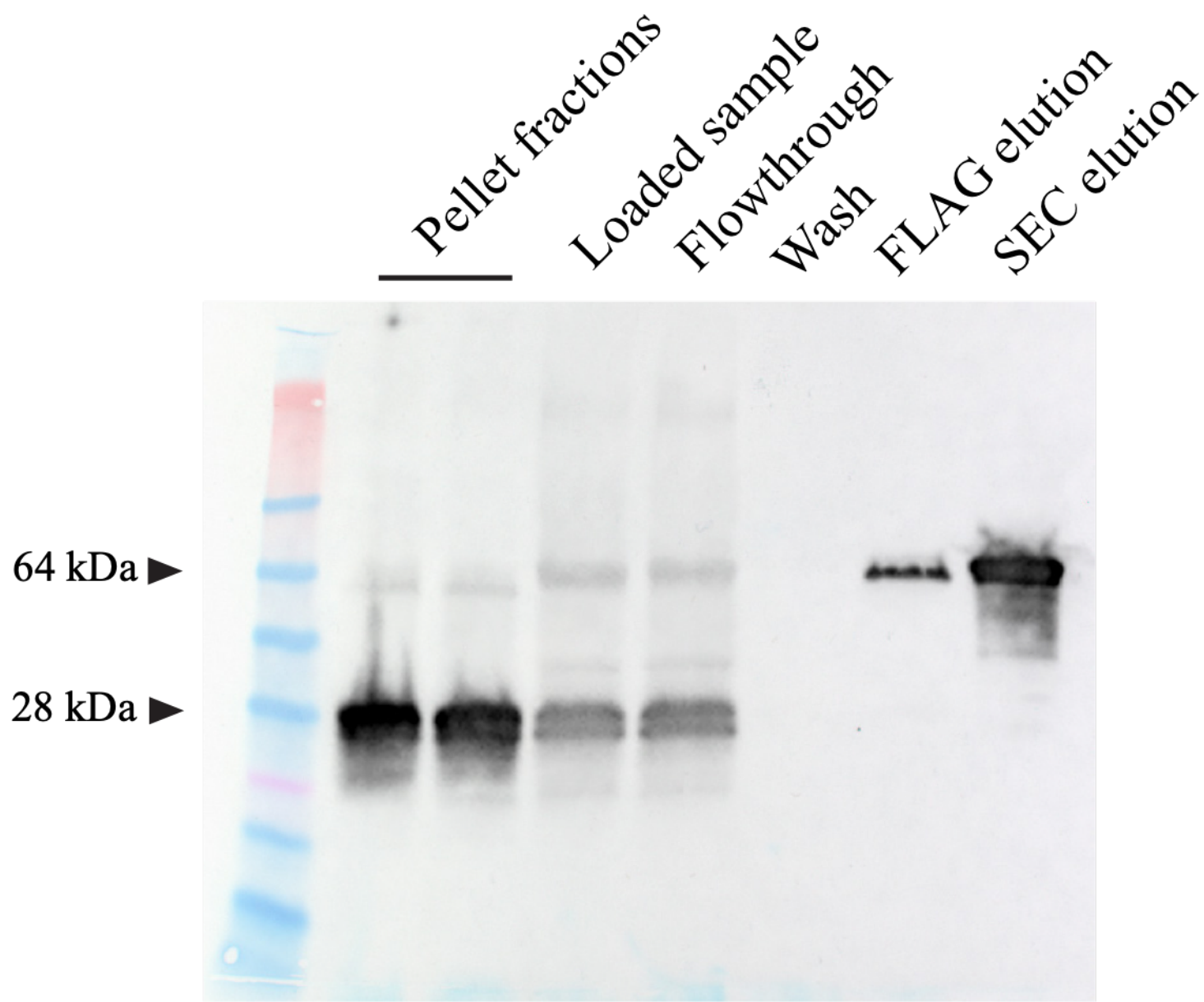

Figure S3: TagR can be purified without detergent.
(A) SDS-PAGE gel of TagR purification from *P. aeruginosa* using anti-FLAG Co-IP followed by SEC. (B) Western blot using anti-TagR antibody of TagR purification from *P. aeruginosa* using anti-FLAG Co-IP followed by SEC.

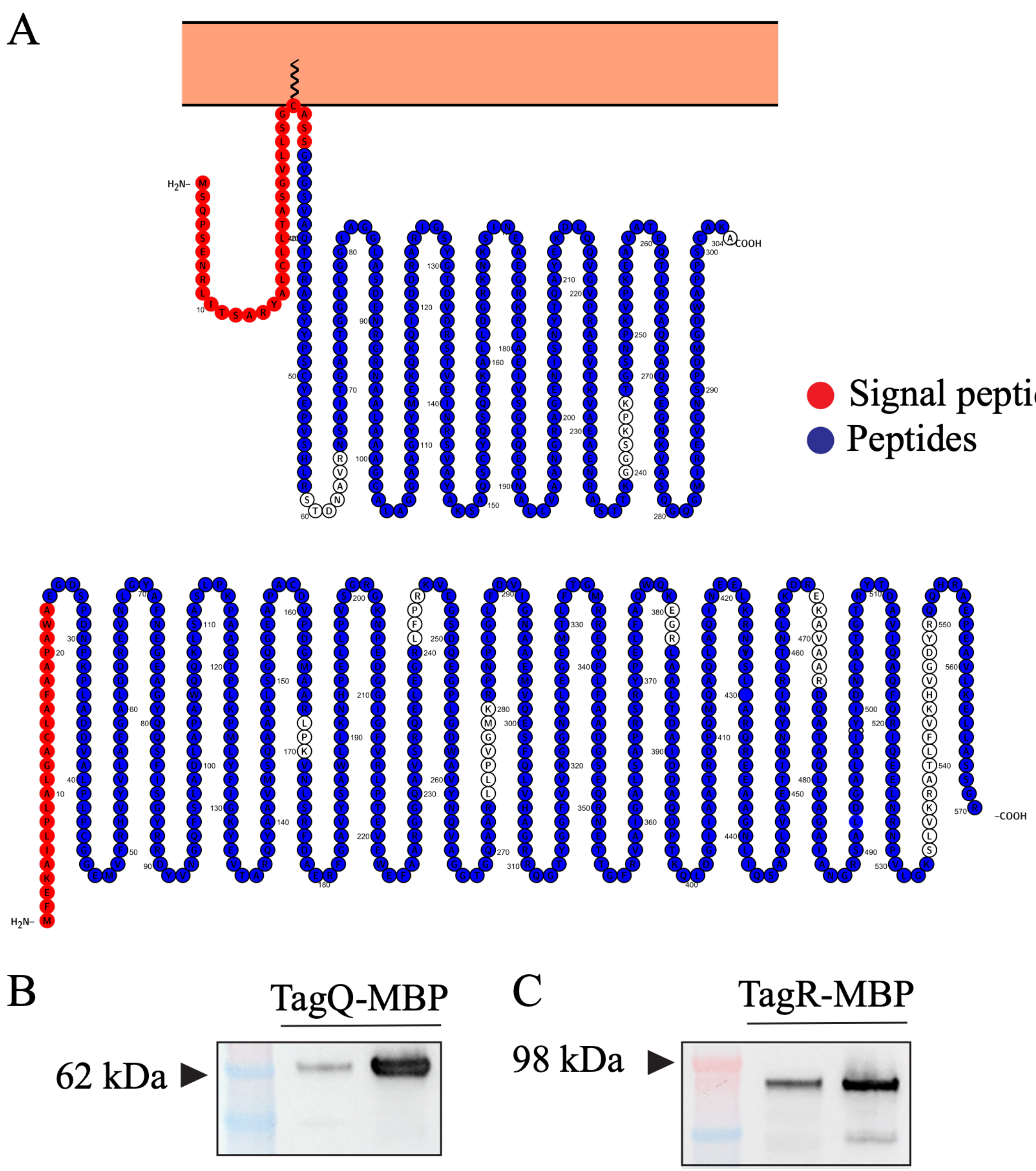

Figure S4: Purification and MS analysis of TagQ and TagR constructs from *E. coli.* (A) Graphical representation of TagQ (top) and TagR (bottom) peptides identified by MS analysis. Image by Protter (http://wlab.ethz.ch/protter). (B) Western blot using anti-TagQ antibody of TagQ linked to MBP (42.5 kDa) expressed in *E. coli*. (C) Western blot using anti-TagR antibody of TagR linked to MBP expressed in *E. coli*.

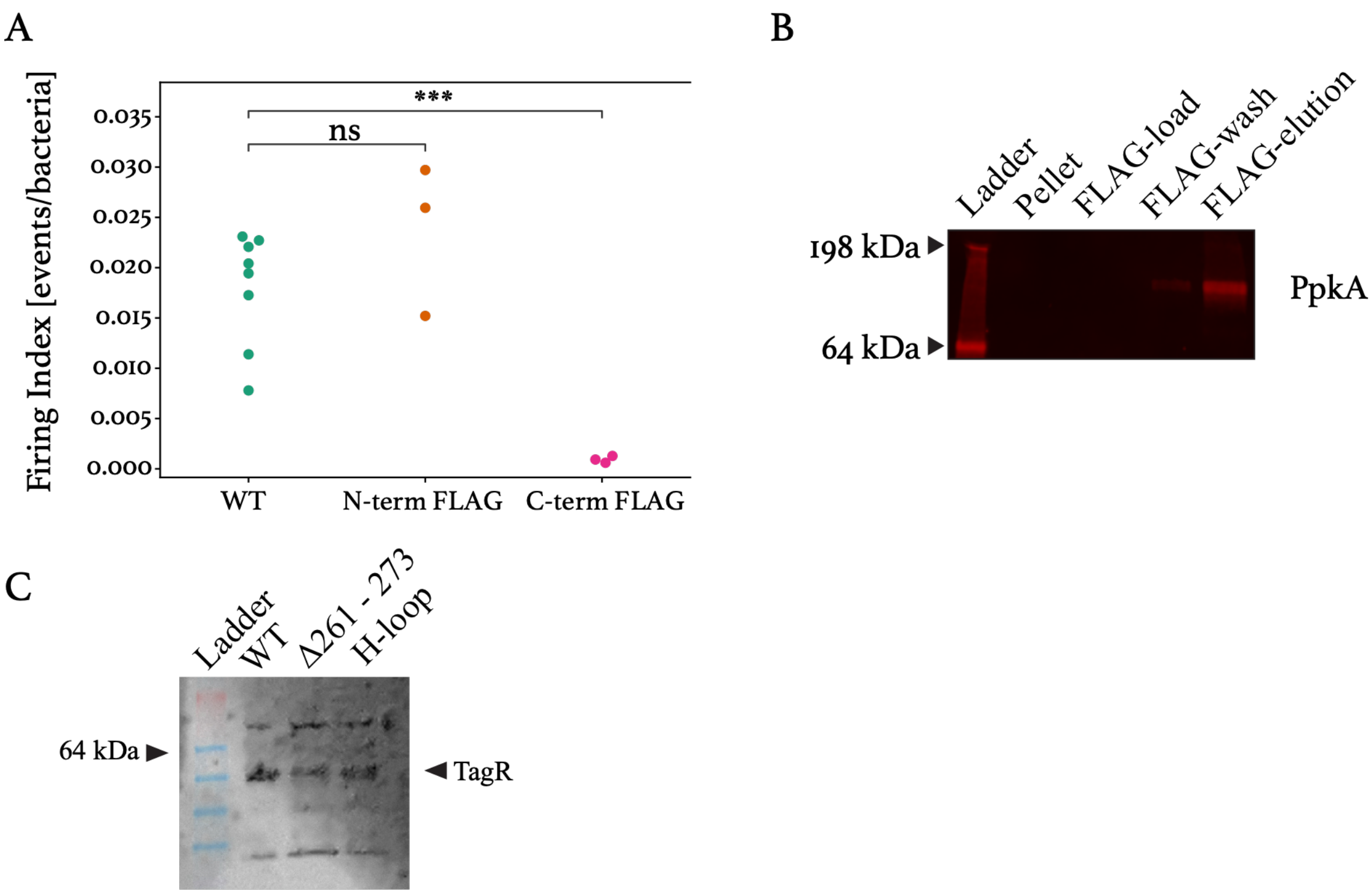

Figure S5: N-terminal FLAG-tag construct of PpkA does not disrupt H1-T6SS activity. (A) Independent t-test statistical analysis of live cell imaging firing events per bacterium on a single frame for WT P. aeruginosa and strains carrying either an N-terminal or C-terminal FLAG tag on PpkA. p-values: $* = p < 0.05$, $** = p < 0.01$, $*** = p < 0.001$, $**** = p < 0.0001$. Two-sided independent student's t-test. All exact p-values available in supplementary table. (B) Western blot using anti-FLAG antibody showing the presence of a band corresponding to the size of PpkA in the FLAG column elution fraction. (C) Western blot using anti-TagR antibody of lysed cells showing the presence of TagR in WT as well as TagR mutant strains.

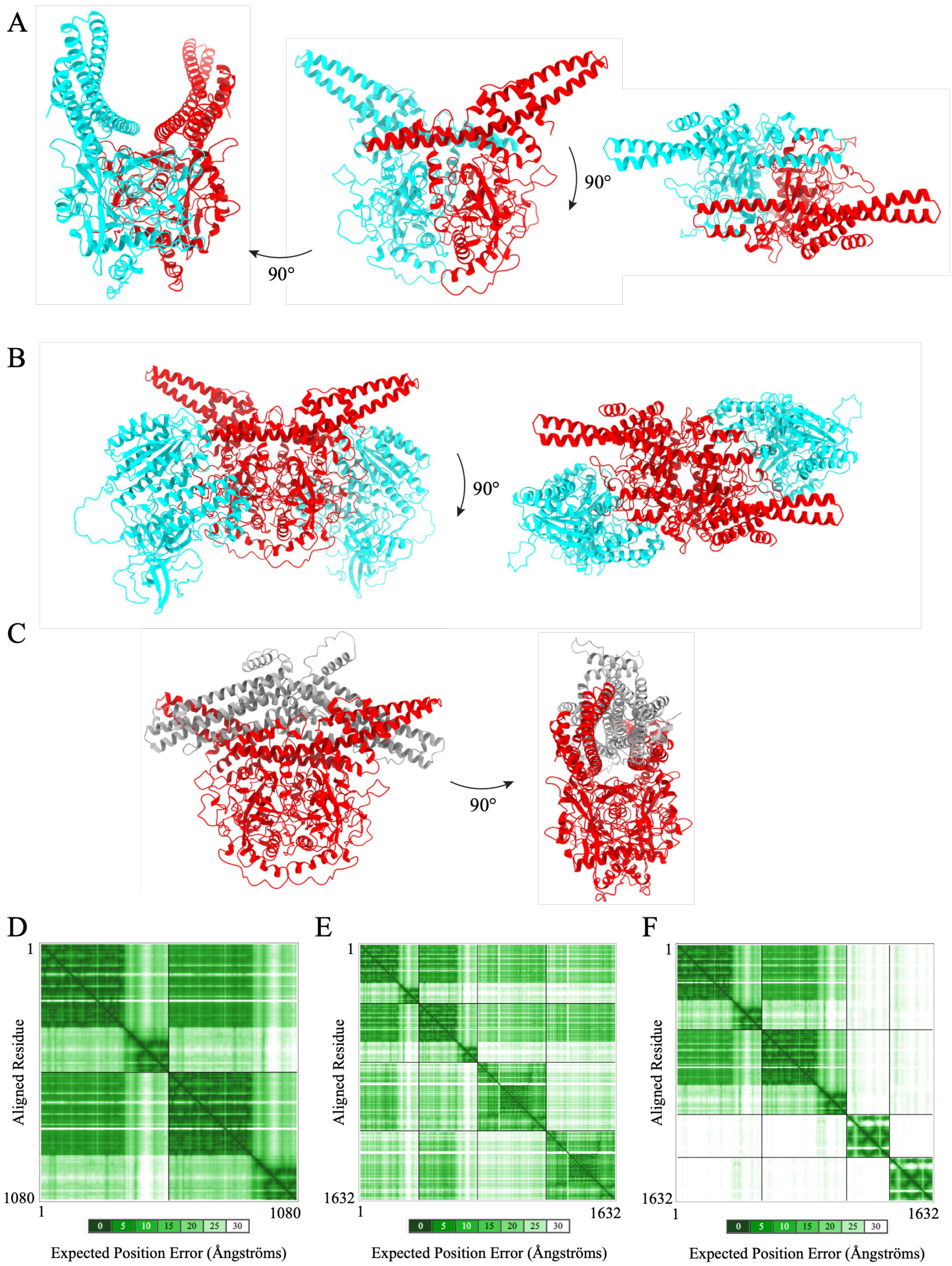

Figure S6: Alphafold3 modeling of TagR dimer, TagR-PpkA and TagR-TagQ complexes. (A) AF3 model of TagR dimer (coloured by chain) showing a symmetrical complex. (B) AF3 model of TagR (red) - PpkA (cyan) complex. (C) AF3 model of TagR (Red) - TagQ (Grey) complex. (D) Predicted aligned error plot of TagR dimer model. ipTM = 0.71 pTM=0.73 (E) Predicted aligned error plot of TagR-PpkA model. ipTM = 0.0.64 pTM=0.67 (F) Predicted aligned error plot of TagR-TagQ model. ipTM = 0.43 pTM=0.55

Table S1: Selected top hits of TagQ-FLAG pulldown

Top 50 significant peptide hits of TagQ-FLAG pulldown, ordered by p-value.

Gene	Log2(fold change)	#-Log10(pvalue)
TagR	6.451211	20
TagQ	1.5824425	8.530663
Q9I4Z7	-0.000000025	5.2606697
Q9HWD0	-0.000000015	4.7861257
Q9HYT5	0.000000021	3.6835632
P00441	4.5849624	3.4873233
Q9HXN2	0.3169925	3.468761
Q9HWF2	-0.000000005	3.3886368
P02768	1.9385995	3.181503
G3XD11	0.00000002	3.179836
Q9HXU9	0.000000015	3.1277807
P05384	0.00000002	3.0541983
P00282	1.9818528	2.9560132
Q9HW32	0.000000025	2.9149306
Q9HXU8	0.000000045	2.8096516
Q9HTN2	3.5849626	2.798351
Q9I3D2	0.000000003	2.6486092
Q9HT18	0.000000038	2.64576
Q9I5Z9	-0.000000083	2.563341
Q9HUV8	0.4169925	2.4822187
Q9HWC5	-0.000000008	2.464099
Q9HVS6	0.000000003	2.461803
Q9HZJ3	2.5443206	2.4502695
Q9I402	1.7900769	2.4129593
P57668	0.000000011	2.4060795
Q9HVX4	0.000000004	2.2735078
Q9I5Q3	2.7548876	2.228503
Q9HX33	2.4594316	2.2083852
Q9I2P8	0.0000004	2.1543941
Q9HVY9	-0.000000011	2.0847504
Q9HXR3	0.000000044	2.0102012
Q9I4H8	-0.000000021	1.9953524
Q9HZJ5	3.9068906	1.9922616
Q9I612	0.2485427	1.9654273
Q9HZP6	0.000000025	1.9650067
Q9HZA6	0.000000004	1.9394104
Q9I5Z0	1.9696264	1.934105
Q9HWF8	-1.4150375	1.9297379
Q9HUM2	0.000000021	1.8773701
Q9I0J7	0.2807355	1.8556089
Q9HWD1	0.000000017	1.8398436
Q9I4K8	0.3807355	1.831496
Q9HZX9	-0.000000049	1.8095071
G3XD94	0.000000013	1.7626027
Q9I742	1.7813597	1.7462195
P30720	0.000000009	1.727483
Q9HXR4	2.3785117	1.7268952
Q9HWF1	-0.000000007	1.7015042
G3XDA5	2.0874627	1.694522

Table S2: Top hits of TagT-FLAG and PpkA-FLAG pulldown

All significant peptide hits of TagT-FLAG and PpkA-FLAG pulldown experiments.

Bait Protein	Prey Protein	SaintScore
tagT-FLAG	TagS	1
tagT-FLAG	TagT	1
ppkA-FLAG	PpkA	0.99

Table S3: Analysis of selected TagT-TID hits

Analysis of MS peptide hits for selected proteins of the TagT-TID labeling experiment.

GeneName	Unique Peptide Count	log2FC_Untreated vs PB	qvalue_Untreated vs PB
hcp1	14	-0.197236808	0.603693065
tssB1	13	-0.011953792	0.983545089
fha1	25	0.25763841	0.37978754
pppA	14	0.026792139	0.953998259
ppkA	36	0.014528332	0.976237054
TagT	11	0.098108334	0.852958782
TagS	9	-0.013972399	0.983545089
TagR	29	-0.068190642	0.796921178
TagQ	24	-0.197107787	0.685936721
clpV1	34	0.013465182	0.976237054
vgrG1b	27	0.001015296	0.997178545
vgrG1a	24	0.017056377	0.976024579

Table S4: Cryo-EM data collection, refinement and validation statistics of TagQR

	TagQR (EMPIAR-13668)
**Data collection and processing**	
Microscope	Titan Krios
Detector	K2 summit
Magnification	165,000x
Voltage (kV)	300
Electron exposure (e–/Å^2)	51
Defocus range (μm)	0.8 – 2.5
Pixel size (Å)	0.82
Movies	6,336
Symmetry imposed	C1 / none
Initial particle images (no.)	2,307,696
Final particle images (no.)	515,798
Map resolution (Å)	Not determined
