# Supplementary material for: Membrane damage induces TagQ/TagR complex-mediated H1-T6SS assembly in Pseudomonas aeruginosa
Source: Nat Commun. 2026 Sep 28;17:11187. doi: 10.1038/s41467-026-78112-9 (PMC13713981; doi:10.1038/s41467-026-78112-9)
Supplement: Supplementary file 4 [file 41467_2026_78112_MOESM2_ESM.pdf]

Description of Additional Supplementary Files

Supplementary Data 1: Coding pipeline for image analysis: environment .yml file

Supplementary Data 2: Coding pipeline for image analysis: batch .ipynb file

Supplementary Data 3: Coding pipeline for image analysis: cells detection .ipynb file

Supplementary Data 4: Coding pipeline for image analysis: data plot .ipynb file

Supplementary Data 5: Coding pipeline for image analysis: spot detection .ipynb file

Supplementary Data 6: Coding pipeline for image analysis: utils .py file

Supplementary Data 7: P-values for microscopy analysis provided as an Excel file

Supplementary Data 8: List of Primers used in this study provided as an Excel file

Supplementary Data 9: List of Strains and plasmids used in this study provided as an Excel file

Supplementary Data 10: Alphafold models generated for this study

Supplementary Video 1: H1- T6SS assembly in natively tagged strains. (1) C-terminal TagR-FLAG construct (*ΔretS tssB1-mNG tagR-FLAG*) imaged without treatment. (2) C-terminal TagRFLAG construct (*ΔretS tssB1- mNG tagR-FLAG*) imaged after treatment with 20 µg/ml Polymyxin B. (3) C-terminal TagQ-FLAG construct (*ΔretS tssB1-mNG tagQ-FLAG*) imaged without treatment. (4) C-terminal TagQ-FLAG construct (*ΔretS tssB1-mNG tagQ-FLAG*) imaged after treatment with 20 µg/ml Polymyxin B. (5) N-terminal TagT-FLAG construct (*ΔretS tssB1-mNG tagT-FLAG*) imaged without treatment. (6) N-terminal PpkA-FLAG construct (*ΔretS tssB1-mNG ppkA-FLAG*) imaged without treatment. (7) N-terminal TagTTurboID construct (*ΔretS tssB1-mNG tagt-TurboID)* imaged without treatment. GFP channel shown, 30 frames recorded every 3 s, contrast enhanced, 2x scaling in x and y axis.

Supplementary Video 2: TagT K44A mutant activity is similar to parental strain. (1) TagT K44A mutant (*ΔretS tssB1-mNG tagT K44A*) imaged without treatment. (2) TagT K44A mutant strain imaged after treatment with 20 µg/ml Polymyxin B. GFP channel shown, 30 frames recorded every 3 s, contrast enhanced, 2x scaling in x and y axis.

Supplementary Video 3: Treatment of P. aeruginosa cells with membrane damage inducing molecules. (1) Parental strain cells (*ΔretS tssB1-mNG*) without treatment. (2) Parental strain cells treated with 2 mM EDTA. (3) Parental strain cells treated with 20 µg/ml Polymyxin B. (4) Parental strain cells treated with 40 µg/ml PMBN. (5) Parental strain cells treated with 40 µg/ml melittin. GFP channel shown, 30 frames recorded every 3 s, contrast enhanced, 2x scaling in x and y axis.
